# Modelling the spread of two successive SIR epidemics on a configuration model network

**DOI:** 10.1007/s00285-025-02207-y

**Published:** 2025-04-23

**Authors:** Frank Ball, Abid Ali Lashari, David Sirl, Pieter Trapman

**Affiliations:** 1 School of Mathematical Sciences, University of Nottingham, University Park, Nottingham, NG7 2RD UK; 2 Department of Mathematics, Stockholm University, Stockholm, 10691 Sweden; 3 Unit of Occupational Medicine, Institute of Environmental Medicine, Karolinska Institutet, Stockholm, 11365 Sweden; 4 Bernoulli Institute, University of Groningen, Nijenborgh 9, Groningen, 9747 AG The Netherlands

**Keywords:** Subsequent SIR epidemics, Reed-Frost model, Percolation, Multi-type branching processes, 05C80, 60J80, 60J85, 60K35, 92D30

## Abstract

We present a stochastic model for two successive SIR (Susceptible $\rightarrow$ Infectious $\rightarrow$ Recovered) epidemics in the same network structured population. Individuals infected during the first epidemic might have (partial) immunity for the second one. The first epidemic is analysed through a bond percolation model, while the second epidemic is approximated by a three-type branching process in which the types of individuals depend on their position in the percolation clusters used for the first epidemic. This branching process approximation enables us to calculate, in the large population limit and conditional upon a large outbreak in the first epidemic, a threshold parameter and the probability of a large outbreak for the second epidemic. A second branching process approximation enables us to calculate the fraction of the population that are infected by such a second large outbreak. We illustrate our results through some specific cases which have appeared previously in the literature and show that our asymptotic results give good approximations for finite populations.

## Introduction

In the first half of 2020 many countries and regions experienced an outbreak of the severe acute respiratory syndrome coronavirus 2 (SARS-CoV-2) pandemic, which causes coronavirus disease 2019 (COVID-19). It took several months of often heavy restrictions for many countries to reduce the prevalence to low levels. After this, many restrictions were relaxed and a second wave of the pandemic hit those countries and regions, spreading among people who were either still susceptible or had become susceptible again after the first wave. Some other diseases, such as influenza, follow seasonal patterns, and often leave part of the population which was infected in one outbreak (partially) immune to an epidemic in the next year. For yet other diseases, having been infected in the past or currently being infected with one disease might give increased susceptibility for the second disease (see e.g. the introductions of Moore et al. (2018) and Bansal and Meyers (2012) and references therein).

The modelling of this kind of interacting stochastic epidemics on (social) networks has been the subject of some recent epidemiological research. Most models assume that the second epidemic spreads after the first epidemic has finished spreading, so the only relevant information from the first epidemic is which individuals were infected during the epidemic, not when they got infected. In this paper we study the case in which two epidemics spread successively in a single population modelled by a configuration model network (e.g. (Durrett (2007), Chapter 3) or (van der Hofstad (2016), Chapter 7)) in which individuals are represented by vertices and relationships which are relevant for the spread of the disease by edges. We assume that a first disease spreads through the network as an SIR (Susceptible $\rightarrow$ Infectious $\rightarrow$ Recovered) epidemic, which causes a major outbreak infecting a nonnegligible fraction of the population. The spread of this epidemic is modelled using bond percolation (see e.g. Grimmett (1999); Kuulasmaa (1982); Newman (2002) and references therein). The connected percolation cluster of vertices of the initial infected vertex is distributed as the set of individuals that are no longer susceptible at the end of the epidemic (Cox and Durrett 1988). A large outbreak then corresponds to the percolation cluster containing the initial infected vertex, being of the same order as the population size (for a detailed description see Ball et al. (2009); Meester and Trapman (2011)).

The first epidemic continues until there is no infectious individual remaining in the population. After that, the second SIR epidemic spreads through the same network, where the transmission probabilities between given individuals depend on whether or not those individuals were infected in the first epidemic. We assume that the first epidemic results in a large outbreak and obtain a threshold parameter which determines whether a large second epidemic is possible, together with expressions for the probability of a large second epidemic and the fraction of the population that are infected by a large second epidemic. These expressions are asymptotically exact in the limit as the population size $n \rightarrow \infty$, unlike much closely related previous work in this area.

SIR epidemics in a closed population are among the most studied class of stochastic epidemic models. In such models, an individual is in one of three possible states, susceptible (*S*), infected (*I*) and recovered (*R*), and the only possible transitions are from *S* to *I* and from *I* to *R*. A first quantity of interest in the SIR model is the basic reproduction number $R_{0}$ (Diekmann et al. (2012), Chapter 7), which is a threshold parameter that determines whether or not major outbreaks infecting a strictly positive fraction of the population occur with non-zero probability as $n\rightarrow \infty$. It is usually defined as the average number of secondary infections caused by a typical newly-infected individual in the early stages of an epidemic started by one or few initially infectious individuals in an otherwise susceptible population. In line with much of stochastic epidemic theory (Andersson and Britton 2000), one can use branching process approximations to prove a threshold theorem which determines whether or not, in the large population limit, there is a non-zero probability for the epidemic to infect a strictly positive fraction of the population. The branching process is subcritical, critical or supercritical if $R_{0}<1$, $R_{0}=1$ or $R_{0}>1$, respectively. These methods also yield approximations for the probability of a large outbreak (i.e. the probability that a supercritical epidemic will grow large) and, using closely related methods, one can also study final outcome (i.e.  fraction of the population that is infected during an epidemic) properties of such a large outbreak (Ball and Neal 2002; Ball et al. 2009; Britton 2010; Kenah and Miller 2011).

We use similar methods to analyse the second epidemic given that the first epidemic resulted in a large outbreak. We approximate the early spread of such a second epidemic using a three-type branching process in which the types of individuals depend on the first epidemic (or, more precisely, on their position in the corresponding percolation component). This approximation yields a threshold parameter for such a second epidemic and also the approximate probability of a large second epidemic. We use a closely related three-type branching process to obtain an approximation to the fraction of the population that are infected by a large second epidemic. A disadvantage of using bond percolation to analyse the first epidemic is that it requires the infectious period to be constant. Our methodology can be extended to the case when the infectious periods are independent and identically distributed, with an arbitrary but specified distribution. However, the arguments are more complex. For ease of presentation we present our results within the framework of a fixed infectious period. We outline how to extend our analysis, using a four-type approximating branching process, to allow for random infectious periods in the discussion in Sect. 7.

A number of authors have previously considered the spread of more than one epidemic on a network. Newman (2005) studied the existence of a threshold for a second epidemic which spreads on the same configuration model network as a first epidemic, if the individuals that were infected in the first epidemic are completely immune to the second one. Bansal and Meyers (2012) work in the same framework but relax the assumption of the first epidemic giving complete immunity to the second epidemic, so instead it confers partial immunity. However, as we show below, the analysis in Bansal and Meyers (2012) is based on implicit approximations, which are not exact in the large population limit. Newman and Ferrario (2013) consider the case in which only individuals infected during the first epidemic are susceptible to the second epidemic. Moore et al. (2018) consider a similar model on an Erdős-Rényi random graph (see e.g. (Durrett (2007), Chapter 2), (van der Hofstad (2016), Chapters 4 and 5)), but in their model the second epidemic can only infect individuals which are still infectious in the first epidemic. So, in contrast to the models in Newman (2005); Bansal and Meyers (2012); Newman and Ferrario (2013), Moore et al. have to keep track of the spread of the first epidemic as it evolves in time in order to study the spread of the second epidemic. Funk and Jansen (2010) consider the spread of two epidemics in the same population, where the set of connections between individuals are different for the two epidemics and are generated as two possibly dependent configuration models. In their model the first epidemic provides immunity against the second epidemic and they analyse how the spread of the second epidemic is influenced by the dependence between the two networks.

Our model is closely related to the models presented by Newman and Ferrario (2013) and Bansal and Meyers (2012). Bansal and Meyers (2012) present two models involving partial immunity. First, they model *polarized* partial immunity by assuming that a fraction of the individuals infected in the first epidemic are immune to the second epidemic while all other individuals are fully susceptible for the second epidemic (cf. all-or-nothing vaccination in e.g. Halloran et al. (1992)). They consider the number of neighbours (individuals connected by an edge in the configuration model) which are susceptible to the second epidemic, of a uniformly chosen individual among the individuals susceptible for the second epidemic and create a new configuration model of those individuals susceptible to the second epidemic. Using the standard configuration model and bond percolation on it, they derive epidemiological quantities for a second outbreak in the partly immunised population. Following arguments in Newman and Ferrario (2013), we note that this approach leads to an approximation which is *not* exact in the large population limit. This can be seen by considering the individuals infected during the first epidemic. Those vertices necessarily form a connected subgraph. However, in a newly constructed configuration model, they do not need to be all connected. In their second model, Bansal and Meyers (2012) consider *leaky* partial immunity (cf. leaky vaccination in e.g. Halloran et al. (1992)), which reduces both the infectivity and susceptibility by constant factors, which are the same for all individuals infected in the first epidemic. In Bansal and Meyers’ analysis of this model, a two-type bond percolation process is used. As for polarized partial immunity, this leads to a tractable analysis which permits exploration of the properties of the approximating model. However, it too is *not* exact in the large population limit, again because the vertices that are infected in the first epidemic necessarily form a connected subgraph, a property which is lost in the approximation of Bansal and Meyers (2012).

This paper is organised as follows.

- In Sect. 2, we describe our epidemic model. We introduce the configuration model graph/network and the two consecutive SIR epidemics defined on this graph. We describe the polarized and leaky partial immunity models.
- In Sect. 3, we state the main results of our paper in four theorems. Theorems 3.1 and 3.2 give a threshold parameter for a second large outbreak to have strictly positive probability and the computation of the probability of such a large outbreak, respectively. Theorem 3.3 gives the computation of the fraction of the population infected by a large second epidemic. Leaky partial immunity is a special case of our more general model for the second epidemic, so these three theorems apply to that model. Theorem 3.4 gives equivalent results to the first three theorems but for polarized partial immunity, which does not fit exactly our framework. Computation of the threshold parameter and large outbreak probability for the second epidemic is the same as before but computation of the fraction infected by a large epidemic needs modification.
- In Sect. 4, we analyse three examples. The first and second correspond to the modelling of polarized and leaky partial immunity as introduced by Bansal and Meyers (2012). The third concerns the herd immunity threshold after a large first epidemic when individuals who are infected by it are immune to the second epidemic.
- In Sect. 5, we show through numerical simulations that our asymptotic results perform well in finite networks. Furthermore, we explore analytic model behaviour for various models of partial immunity.
- In Sect. 6, we provide the proofs of the main theorems. To do this we first present an approximating three-type branching process for the second epidemic and compute its mean offspring matrix and its probability of survival, which corresponds to the probability of a large outbreak in the second epidemic. Using another approximating three-type branching process, we also give a recipe to compute the fraction of the population infected in such a large outbreak.
- In Sect. 7, we discuss some possible extensions, including non-constant infectious periods, and other future work.

## The epidemic model

### The configuration model

Formally, we consider $\{E^{(n)};{n \in \mathbb {N}}\}$, a sequence of models for the spread of SIR epidemics on random graphs/networks. The epidemic $E^{(n)}$ spreads on the random graph $\mathcal {G}= \mathcal {G}^{(n)} = (\mathcal {V}^{(n)}, \mathcal {E}^{(n)})$, where the vertex set $\mathcal {V}^{(n)}$ consists of *n* vertices that represent the individuals. The edges in the edge set $\mathcal {E}^{(n)}$ represent connections/relationships between individuals through which infection might transmit. We obtain results for the asymptotic case $n \rightarrow \infty$. In the remainder of the paper we often suppress the superscript (*n*).

The graph $\mathcal {G}$ is generated by (a version of) the configuration model; for a detailed description see (Durrett (2007), Chapter 3) or (van der Hofstad (2016), Chapter 7). We construct $\mathcal {G}$ by assigning an i.i.d. (independent and identically distributed) number of half-edges to each vertex. The number of half-edges assigned to a vertex is called its degree. The degrees are distributed according to an arbitrary but specified discrete random variable *D* having probability mass function

$$\begin{aligned} p_k= \mathbb {P}(D = k), \qquad (k = 0, 1,...). \end{aligned}$$

We assume that $\mathbb {E}[D^{2}]<\infty$. The half-edges are then paired uniformly at random to create the edges in the graph. In this construction some imperfections might occur. If the total number of half edges is odd, we ignore the single leftover half-edge which has no effect on the degree distribution as $n\rightarrow \infty$. Furthermore, it is well-known that the numbers of self-loops (edges from a vertex to itself) and multiple edges between the same pair of vertices are small (the numbers are bounded in expectation as $n \rightarrow \infty$) under our assumption that $\mathbb {E}[D^{2}]<\infty$ (van der Hofstad (2016), p. 230). Moreover, the probability that the graph has no such imperfections is bounded below by a strictly positive constant as $n \rightarrow \infty$, so convergence in probability results may be transferred from epidemics on the constructed graph $\mathcal {G}$ to those on $\mathcal {G}$ conditioned on having no such imperfections (Janson 2014). Therefore we can safely ignore self-loops and merge parallel edges.

In the construction of the configuration model, a half-edge is *k* times as likely to be paired with a half-edge emanating from an individual with degree *k* than with one emanating from an individual with degree 1. Therefore, a typical (i.e. uniformly chosen) neighbour of a typical individual in the network has a size-biased degree distribution (Newman 2002) defined through

2.1 $$\begin{aligned} \tilde{p}_{k}=\mathbb {P}(\tilde{D}=k)=\frac{1}{\mu _{D}}kp_k,\qquad (k=1,2,\cdots ), \end{aligned}$$

where

2.2 $$\begin{aligned} \mu _{D}=\sum _{\ell =0}^{\infty }\ell p_{\ell } \end{aligned}$$

is the expected degree of a vertex.

### The first epidemic

In this subsection we discuss how the first epidemic spreads on a network. For a more extensive discussion and some heuristic and rigorous analyses of that epidemic see e.g. (Durrett (2007), Section 3.5), Andersson (1999); Ball et al. (2009); Kenah and Robins (2007b); Miller (2007); Newman (2002).

The first epidemic is defined as follows. We consider an SIR epidemic on $\mathcal {G}$. Each individual is assumed to be susceptible, infectious or recovered. We say that a vertex is susceptible, infectious or recovered if the individual it represents is in that “infection state”. Throughout we assume that at time 0, one individual chosen uniformly at random from the population becomes infected, while all other individuals are susceptible. Our model and analysis can easily be generalised to other initial conditions, such as having a bounded (as $n \rightarrow \infty$) number of initially infected individuals chosen uniformly at random, or assuming that the initial infectious individual has a specified degree.

Neighbours in the population, i.e. individuals/vertices connected by an edge in $\mathcal {G}$, contact each other according to independent homogeneous Poisson processes with rate $\beta$. If the contact is between a susceptible and an infectious vertex, then the susceptible one immediately becomes infectious; otherwise the contact has no effect. In many papers on stochastic epidemics, infectious individuals stay infectious for a possibly random period distributed as the random variable *L*. However, in order to improve the readability of the analysis in this paper and avoid having to deal with some dependencies (see e.g., Kuulasmaa (1982); Meester and Trapman (2011); Kenah and Miller (2011)), we assume that *L* is constant, i.e. *L* is not random. Without loss of generality we assume that $\mathbb {P}(L=1)=1$ (i.e. we scale time such that the infectious period is one time unit). In Sect. 7, we remark on the model with more general distributions of *L*. In particular we discuss why extending the analysis is not trivial, but still explain how this extension can be made. Because *L* is not random, the first epidemic on the network has the same final size distribution as a corresponding Reed-Frost model (see e.g. (Andersson and Britton (2000), p. 17)). Because $\mathbb {P}(L=1)=1$, any given infected individual makes at least one contact during its infectious period with each of its neighbouring individuals independently and with the same probability,

$$\begin{aligned} p=1-\textrm{e}^{-\beta }. \end{aligned}$$

The first epidemic ends when there is no infectious individual remaining in the population.

We define the basic reproduction number of the first epidemic as

2.3 $$\begin{aligned} R_0^{(1)}= p \mathbb {E}[\tilde{D}-1]=p\left( \mu _{D}+\frac{\sigma ^{2}_{D}}{\mu _{D}}-1\right) , \end{aligned}$$

where $\tilde{D}$ is defined in (2.1), $\mu _D$ in (2.2) and $\sigma ^{2}_{D}=\sum _{\ell =0}^{\infty }\ell ^{2} p_{\ell }-\mu _{D}^{2}$ is the variance of *D*. Note that $\mathbb {E}[\tilde{D}-1]$ is the expected number of susceptible neighbours a typical vertex has just after being infected in the early stages of the epidemic, while each of those susceptible neighbours is infected by this vertex with probability *p*. So, $R_0^{(1)}$ is the expected number of neighbours infected by a typical infected vertex during the early stages of an epidemic. It is well-known (e.g. Newman (2002); Britton (2010)) that this quantity is a threshold parameter for the first epidemic: in the limit as the population size $n \rightarrow \infty$, a large outbreak occurs with strictly positive probability if and only if $R_0^{(1)}>1$.

It follows from branching process approximations, see e.g. (Durrett (2007), Theorem 3.5.1), Kenah and Robins (2007b) or Ball et al. (2009), that both the probability of a large outbreak and, conditioned on a large outbreak, the fraction of the population infected converge (as $n \rightarrow \infty$) to the probability that a well-chosen two-stage Galton-Watson process survives. This probability can be shown to be $1-q$, where the constant *q* is given by

2.4 $$\begin{aligned} q=\mathbb {E}[(1-p+p \tilde{q})^{D}], \end{aligned}$$

with $\tilde{q}$ being given by the smallest positive solution of

2.5 $$\begin{aligned} s=\mathbb {E}[(1-p+ps)^{\tilde{D}-1}]. \end{aligned}$$

Indeed, it is easily checked that $\tilde{q} \in [0,1)$ (and hence $q \in [0,1)$) if and only if $R_0^{(1)}>1$, excluding the pathological case when all vertices have degree 2 and $p=1$. The equality of the probability of a large outbreak and the fraction of the population infected in a large outbreak follows immediately if one uses bond percolation arguments (see e.g. Cox and Durrett (1988); Kenah and Miller (2011); Meester and Trapman (2011) and Sect. 6.1 below) to study the epidemic, and critically depends on the assumption that the infectious period is not random, so the events that a given infectious vertex makes infectious contacts with different neighbours are independent.

### The second epidemic

To analyse the second epidemic we keep track of whether or not vertices were infected in the first epidemic. So, we need four rates $\beta _{00}$, $\beta _{01}$, $\beta _{10}$ and $\beta _{11}$ to denote the rates of infectious contacts between different types of vertices. The parameter $\beta _{00}$ is the rate at which an individual who was not infected during the first epidemic makes infectious contacts with a given neighbour who was also not infected during the first epidemic. Here infectious contacts need not be symmetric and are defined as contacts which would lead to infection if the “contacter” is infectious and the “contactee” is susceptible. Likewise, $\beta _{01}$ is the rate at which an individual who was not infected during the first epidemic makes infectious contacts with a given neighbour who was infected during the first epidemic. The rates $\beta _{10}$ and $\beta _{11}$ are defined similarly. Furthermore, we assume that the infectious period of the disease spreading in the second epidemic for an *i*-individual ($i \in \{0,1\}$) is fixed at length $\ell _i^{(2)}$ and define

2.6 $$\begin{aligned} \pi _{ij} = 1-\textrm{e}^{-\beta _{ij} \ell _i^{(2)}}, \qquad \text{ for } i,j \in \{0,1\}, \end{aligned}$$

that is, $\pi _{ij}$ is the probability that, if it gets infected, an *i*-individual makes infectious contact with a given *j*-neighbour. As for the first epidemic, we assume that the second epidemic starts with one infectious individual chosen uniformly at random from the population. Our model and analysis can easily be generalised to some other initial conditions, such as having a bounded (as $n \rightarrow \infty$) number of initially infected individuals chosen uniformly at random, or assuming that the initial infectious individual has a specified degree.

#### Remark 2.1

It is easy to check that if $\pi _{11}=p'$ for some $p'\in [0,1]$ and $\pi _{10}=\pi _{01}=\pi _{00}= 0$ then our model corresponds to the model in Newman and Ferrario (2013). Moreover, if we take $\pi _{00}=p'$ for some $p'\in [0,1]$ and $\pi _{11}=\pi _{01}=\pi _{10}= 0$ then our model corresponds to the model in Newman (2005). Thus, the models in Newman and Ferrario (2013) and Newman (2005) are special cases of our model.

For our analysis we use in Sect. 6.1 that asymptotically (as $n \rightarrow \infty$) the graph $\mathcal {G}$ is locally tree-like. We analyse the epidemic on a random tree-graph $\mathcal {T}$, where the root of the tree is the initially infected vertex of the second epidemic. The root has degree distribution *D*, while the other vertices in $\mathcal {T}$ have degree distribution $\tilde{D}$. This tree is such that locally the distribution of the “neighbourhood” of the root is similar to the distribution of the neighbourhood of the initially infectious individual (for the second epidemic) in $\mathcal {G}$. The first epidemic on the tree spreads “from infinity”. That is, independently, we colour the edges of $\mathcal {T}$ red with probability *p*, while the other edges of $\mathcal {T}$ are blue. In addition we colour all vertices that are part of an infinite path of red edges in $\mathcal {T}$ red, while all other vertices are blue. The red vertices are infected by the first epidemic. Note that the first epidemic can spread both towards the root and away from the root. We denote the coloured version of $\mathcal {T}$ by $\mathcal {T}_*$. We call an infinite path of red edges that does not contain any pair of siblings (i.e. a path that can be covered while only moving away from the root) a red path of descent (see Fig. 1).

**\footnotesize Figure 1:**
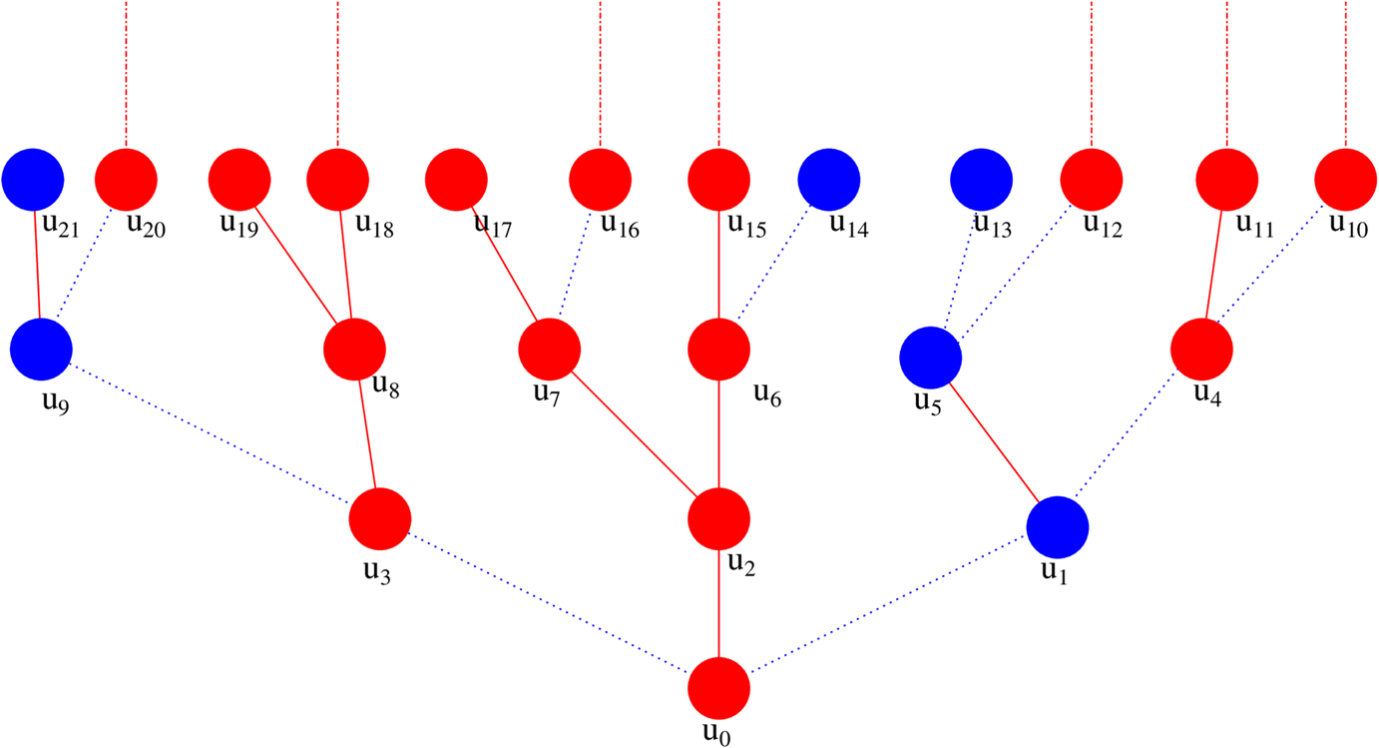
The coloured tree $\mathcal {T}_*$ with root $u_0$. In this network every vertex has degree 3 and each vertex other than $u_0$ has one “parent” and two “children”. The red vertices represent individuals infected in the first epidemic and blue vertices represent uninfected individuals. The solid red edges represent relationships through which the first epidemic would transmit if at least one of the end-vertices were infected in the first epidemic. The dotted blue edges represent relationships through which the first epidemic would not transmit, even if one of the end vertices got infected. The dashed-dotted red lines represent (infinite) red paths of descent in $\mathcal {T}_*$

We now label the vertices in $\mathcal {T}$ by three different types. Vertices that have an (infinite) red path of descent in $\mathcal {T}_*$ (and are necessarily red) are of type 1. Type-2 vertices correspond with red vertices that do not have a red path of descent, while type-3 vertices are blue. Using these three types we show in Sect. 6 that, if we ignore the root, the random tree $\mathcal {T}$ with the three types of vertices is indeed distributed as a three-type Galton-Watson tree (Jagers (1975), Chapter 4). So in Fig. 1, the blue vertices are of type 3, $u_7$, $u_{17}$ and $u_{19}$ are of type 2 and all other depicted red vertices are of type 1.

We study the spread of the second epidemic (which by construction spreads away from the root) on $\mathcal {T}_{*}$, where the infected individuals get the type of their vertex, using a multi-type extension of the spread of epidemics on configuration model graphs (see e.g. (Durrett (2007), Section 3.5)).

### Models for partial immunity

In this subsection we describe the models for polarized and leaky partial immunity as introduced by Bansal and Meyers (2012) and discuss their relationship to the model for the second epidemic in Sect. 2.3. These model serve as illustrative examples of the general results of this paper.

#### Polarized partial immunity

In the polarized partial immunity model, every recovered individual after the first epidemic becomes susceptible to the second epidemic independently with probability $\alpha$, while the other recovered individuals stay immune. Note that this model does not fit completely the framework in Sect. 2.3. Indeed, the susceptibility of an individual to the second epidemic depends not only on whether or not it was infected by the first epidemic, but also on whether it is rendered immune to the second epidemic if it was infected by the first epidemic. The latter is random unless $\alpha =0$ or $\alpha =1$. However, we show in Sect. 3.4 how our framework can be used to determine the asymptotic probability of a large second epidemic and how it can be adapted to determine the asymptotic final size of such an epidemic.

#### Leaky partial immunity

Recall that under leaky partial immunity all individuals infected by the first epidemic are affected identically in terms of the second epidemic; they have reduced susceptibility and also reduced infectivity if they are infected. Bansal and Meyers (2012) model this by assuming that the probability of transmission per susceptible-infectious neighbour pair is reduced by a factor $\alpha$ if one of the pair was infected by the first epidemic and by a factor $\alpha ^2$ if both were infected by the first epidemic. We generalise this by having factors $\alpha _S$ and $\alpha _I$ for susceptibility and infectivity, respectively. This generalisation fits our general theory with

2.7 $$\begin{aligned} \pi _{00}=p', \pi _{01}=\alpha _S p', \pi _{10}=\alpha _I p' \text { and } \pi _{11}=\alpha _S \alpha _I p', \end{aligned}$$

where $p'$ is the probability that in the second epidemic an infective infects a given susceptible neighbour who was not infected by the first epidemic, which can be different from *p*, the infection probability in the first epidemic.

An alternative, which aligns more closely with models for vaccine action is to assume that these reductions are in terms of changed probability of transmission per contact between a susceptible and infectious individual, depending on whether the individuals involved have been infected before. To model this, give individuals in the second epidemic a further typing; specifically call individuals that were infected by the first epidemic type *V* and those that were not infected type *U*. (This labelling reflects the similarity between immunity conferred by prior infection to that conferred by vaccination.) Then, in the second epidemic, each contact between an infective and a type-*V* susceptible leads to the infection of the latter independently with probability $\alpha _S$, and the rate at which a type-*V* infective contacts its neighbours is a factor $\alpha _I$ lower than the corresponding rate for a type-*U* infectious. This implies that the $\beta _{ij}$s defined just before (2.6) take the form $\beta _{00}=\beta ', \beta _{01}=\alpha _S\beta ', \beta _{10}=\alpha _I\beta '$ and $\beta _{11}=\alpha _S \alpha _I \beta '$, whence (2.6) now yields

2.8 $$\begin{aligned} \pi _{00}\!=\! 1-\textrm{e}^{-\beta '\ell _0^{(2)}}, \pi _{01}\!=\!1-\textrm{e}^{-\beta '\alpha _{S}\ell _0^{(2)}}, \pi _{10}\!=\!1-\textrm{e}^{-\beta '\alpha _{I}\ell _1^{(2)}} \text { and } \pi _{11}=1-\textrm{e}^{-\beta '\alpha _{S}\alpha _{I}\ell _1^{(2)}}.\nonumber \\ \end{aligned}$$

Note that the contact rate $\beta '$ between individuals not infected by the first epidemic need not be the same as the contact rate $\beta$ in the first epidemic.

##### Remark 2.2

We note that here (and in Bansal and Meyers (2012)) the term leaky has a slightly different meaning than it usually has in vaccine response models, where it is usually assumed that infectivity is not affected by the vaccine (i.e. $\alpha _I=1$). Our model for leaky partial immunity is analogous to the non-random vaccine response of Ball and Lyne (2006), which is a special case of the vaccine action model introduced by Becker and Starczak (1998).

## Results

In this section we present the main results of the paper, which provide for the second epidemic (i) a threshold parameter which determines whether a large outbreak is possible, (ii) the probability that a large outbreak occurs and (iii) the fraction of the population that are infected by a large second outbreak. These results are all conditional upon a large first epidemic, which is assumed implicitly throughout this section. Strictly speaking, we prove the theorems for the second epidemic on the approximating coloured tree as described in Sect. 2.3, while we provide a heuristic justification for why using the coloured tree will lead to the corresponding results on a configuration model. Making this justification fully rigorous requires a technical proof similar to proofs in e.g. Ball et al. (2009, 2014), which is beyond the scope of this paper.

Many of the results are expressed in terms of the probability generating functions (PGFs) $f_D$ and $f_{\tilde{D}-1}$ of *D* and $\tilde{D}-1$, respectively. For $x \in [0,1]$,

$$\begin{aligned} f_D(x)=\mathbb {E}[x^D]=\sum _{k=0}^{\infty } p_k x^k \qquad \text {and}\qquad f_{\tilde{D}-1}(x)=\sum _{k=1}^{\infty } \tilde{p}_kx^{k-1}. \end{aligned}$$

Note that

3.1 $$\begin{aligned} f_{\tilde{D}-1}(x)=\mu _D^{-1}f_D^{\prime }(x) \quad (x \in [0,1]), \end{aligned}$$

where $^\prime$ denotes derivative. This representation is useful, because for many probability distributions the PGFs have a convenient form.

### Reproduction number for second epidemic

Recall the coloured tree $\mathcal {T}$ introduced in Sect. 2.3. It is fruitful to introduce a further labelling of vertices. Specifically, type-1 vertices are labelled *A* and type-2 and type-3 vertices are labelled *B*. Thus vertices labelled *A* have a (infinite) red path of descent and vertices labelled *B* do not. For $i, j \in \{A, B\}$, let $\mu _{ij}$ be the mean number of children (cf. Fig. 1) of a typical (non-root) label-*i* vertex in $\mathcal {T}$ that have label *j*. We show in Sect. 6.3 that

3.2 $$\begin{aligned} \mu _{AA}&=\frac{1}{\mu _D} \left( f_D''(1) -(1-p)f_D''(1-p+p \tilde{q})\right) ,\end{aligned}$$

3.3 $$\begin{aligned} \mu _{AB}&= \frac{1}{\mu _D} \left( f_D''(1) -f_D''(1-p+p \tilde{q})\right) ,\end{aligned}$$

3.4 $$\begin{aligned} \mu _{BA}&= \frac{(1-p)(1-\tilde{q})}{\tilde{q}} \frac{1}{\mu _D} f_D''(1-p+p \tilde{q}),\end{aligned}$$

3.5 $$\begin{aligned} \mu _{BB}&= \frac{1}{\mu _D} f_D''(1-p+p \tilde{q}) , \end{aligned}$$

where $\tilde{q}$ is defined as in (2.5).

As indicated in Sect. 2.3, we approximate the spread of the second epidemic by a three-type Galton-Watson process, $\mathcal {B}^{(2)}_F$, which describes the spread of the second epidemic on $\mathcal {T}$. Note that a label-*B* child of a red individual is type 2 if and only if it is infected by its parent in the first epidemic, and all label-*B* children of a blue individual are necessarily type 3. It follows that the offspring mean matrix *M* for a non-initial generation of $\mathcal {B}^{(2)}_F$ is given by

3.6 $$\begin{aligned} M=\begin{pmatrix} \pi _{11}\mu _{AA} & p\,\pi _{11}\mu _{AB} & (1-p)\pi _{10}\mu _{AB} \\ \pi _{11}\mu _{BA} & p\,\pi _{11}\mu _{BB} & (1-p)\pi _{10}\mu _{BB} \\ \pi _{01}\mu _{BA} & 0 & \pi _{00}\mu _{BB} \end{pmatrix}, \end{aligned}$$

where for $i,j \in \{0,1\}$, $\pi _{ij}$ is defined in (2.6) and *p* is the per pair of neighbours infection probability of the first epidemic. We are now ready to formulate our first main result, which follows using standard properties of multitype Galton-Watson processes (e.g. (Jagers (1975), Theorem 4.2.2)).

#### Theorem 3.1

Consider the model defined in Sect. 2. Let $R_0^{(2)}$ be the dominant eigenvalue of the matrix *M*. The second epidemic has a strictly positive probability to be major if and only if $R_0^{(2)} >1$.

### Probability of a large second epidemic

The next result concerns the probability of a major outbreak of the second epidemic, which in the limit as $n \rightarrow \infty$ is given by the survival probability of the branching process $\mathcal {B}^{(2)}_F$. Note that the offspring distribution of the initial individual in $\mathcal {B}^{(2)}_F$, which corresponds to the root of $\mathcal {T}$, is usually different from that of subsequent individuals. (Recall that the root has degree distribution *D*, whereas all other vertices in $\mathcal {T}$ have degree distribution $\tilde{D}$.) Note that the root has type 1 if and only if it was infected in the first epidemic, otherwise it has type 3. Thus the probability that the root has type 1 is 1-*q*, where *q* is given by (2.4).

Let $\textbf{s}=(s_{1},s_{2},s_{3})\in [0,1]^3$. For $i \in \{1,3\}$, let $f_i(\textbf{s})$ be the PGF of the joint offspring distribution of the initial individual in $\mathcal {B}^{(2)}_F$, given that individual has type *i*. For $i \in \{1,2,3\}$, let $\tilde{f}_i(\textbf{s})$ be the PGF of the joint offspring distribution of a type-*i* individual in a non-initial generation of $\mathcal {B}^{(2)}_F$. We show in Sect. 6.4 that these PGFs are as follows, where we use, for $i,j \in \{1,2\}$, the notation

3.7 $$\begin{aligned} t_{ij}(s_j)&= 1-\pi _{11}+\pi _{11}s_j,\nonumber \\ t_{i3}(s_3)&= 1-\pi _{10}+\pi _{10}s_3,\nonumber \\ t_{31}(s_1)&= 1-\pi _{01}+\pi _{01}s_1,\nonumber \\ t_{32}(s_2)&= 0,\nonumber \\ t_{33}(s_{3})&= 1-\pi _{00}+\pi _{00}s_3. \end{aligned}$$

For $$i\in \{1,3\}$$ and $$\textbf{s} \in [0,1]^3$$,

3.8 $$\begin{aligned} f_{i}(\textbf{s}) = f_{\textbf{Y}_i}(t_{i1}(s_1),t_{i2}(s_2),t_{i3}(s_3)), \end{aligned}$$

where $f_{\textbf{Y}_i}(\textbf{s})$ $(i \in \{1,3\})$ is given by

3.9 $$\begin{aligned} f_{\textbf{Y}_1}({\textbf {s}})&=\frac{1}{1-q}f_{D}(\left( 1-\tilde{q})s_1+\tilde{q}(p s_2+(1-p)s_3\right) )\nonumber \\&\qquad -\frac{1}{1-q}f_{D}(\left( 1-p)(1-\tilde{q})s_1+\tilde{q}(p s_2+(1-p)s_3\right) ), \end{aligned}$$

3.10 $$\begin{aligned} f_{\textbf{Y}_3}(\textbf{s})&= \frac{1}{q}\displaystyle f_{D}\left( (1-p)(1-\tilde{q})s_1+\tilde{q}s_3\right) . \end{aligned}$$

For $$i \in \{1,2,3\}$$ and $$\textbf{s} \in [0,1]^3$$,

3.11 $$\begin{aligned} \tilde{f}_{i}(\textbf{s})=f_{\tilde{\textbf{Y}}_i}(t_{i1}(s_1),t_{i2}(s_2),t_{i3}(s_3)), \end{aligned}$$

where

3.12 $$\begin{aligned} f_{\tilde{\textbf{Y}}_1}(\textbf{s}) =&\frac{1}{1-\tilde{q}}\displaystyle f_{\tilde{D}-1}((1-\tilde{q})s_1+\tilde{q}(p s_2+(1-p)s_3)) \nonumber \\&- \frac{1}{1-\tilde{q}} \displaystyle f_{\tilde{D}-1}((1-p)(1-\tilde{q})s_1+\tilde{q}(p s_2+(1-p)s_3)), \end{aligned}$$

3.13 $$\begin{aligned} f_{\tilde{\textbf{Y}}_2}(\textbf{s}) =&\frac{1}{\tilde{q}} \displaystyle f_{\tilde{D}-1}\left( (1-p)(1-\tilde{q})s_1+\tilde{q}(p s_2+(1-p) s_3)\right) , \end{aligned}$$

3.14 $$\begin{aligned} f_{\tilde{\textbf{Y}}_3}(\textbf{s}) =&\frac{1}{\tilde{q}}\displaystyle f_{\tilde{D}-1}\left( (1-p)(1-\tilde{q})s_{1}+\tilde{q}s_{3}\right) . \end{aligned}$$

#### Theorem 3.2

Consider the model defined in Sect. 2. Assume that $$R_0^{(2)}>1$$ and that the initial infected individual for the second epidemic is chosen uniformly at random. Let $$\varvec{\xi }=(\xi _{1},\xi _{2},\xi _{3})$$ be the unique solution in $$[0,1)^{3}$$ of the system of equations

$$\begin{aligned} \xi _1&= \tilde{f}_1(\varvec{\xi }),\\ \xi _2&= \tilde{f}_2(\varvec{\xi }),\\ \xi _3&= \tilde{f}_3(\varvec{\xi }). \end{aligned}$$

Then the probability of a large outbreak is, asymptotically as $n \rightarrow \infty$, given by

3.15 $$\begin{aligned} 1-(1-q) f_{1}(\varvec{\xi })- q f_{3}(\varvec{\xi }). \end{aligned}$$

Further, the asymptotic probability of a large outbreak is $1-f_{1}(\varvec{\xi })$ if the initial infective was infected in the first epidemic and $1-f_{3}(\varvec{\xi })$ if it was not.

To understand Theorem 3.2, consider first the branching process, $\tilde{\mathcal {B}}^{(2)}_F$, defined analogously to $\mathcal {B}^{(2)}_F$ but starting from a child of the root in $\mathcal {T}$. Thus the offspring distributions are the same for all generations of $\tilde{\mathcal {B}}^{(2)}_F$. For $i \in \{1,2,3\}$, the probability that $\tilde{\mathcal {B}}^{(2)}_F$ goes extinct given it starts with a single individual having type *i* is $\xi _i$; see e.g. (Jagers (1975), Theorem 4.2.2). The branching process $\mathcal {B}^{(2)}_F$ goes extinct if and only if the branching processes starting from each of the children of the initial individual go extinct. Taking expectations with respect to the numbers of individuals of the three types in the first generation of $\mathcal {B}^{(2)}_F$ yields that, for $i \in \{1,3\}$, the probability $\mathcal {B}^{(2)}_F$ goes extinct, given the initial individual has type *i*, is $f_{i}(\varvec{\xi })$. Hence, the asymptotic probability of a large outbreak is given by $1-f_{1}(\varvec{\xi })$ if the initial infective (in the second epidemic) was infected in the first epidemic and by $1-f_{3}(\varvec{\xi })$ otherwise. The unconditional asymptotic probability of a large outbreak is given by (3.15), since the root of $\mathcal {T}$ is infected in the first epidemic with probability $1-q$.

### Size of large second epidemic

For the model defined in Sect. 2 we also obtain results for the fraction of vertices that are ultimately infected by a large second epidemic. The key tool here is the notion of a *susceptibility set*. This is explained in Sect. 6.5 but briefly the idea is as follows. To determine whether an individual, $i_*$ say, is infected by the second epidemic, we first determine which of $i_*$’s neighbours in $\mathcal {G}$ would infect $i_*$ if they were infected by the second epidemic. Next for each such neighbour, $j_*$ say, we determine which of $j_*$’s neighbours in $\mathcal {G}\setminus \{i_*\}$ would infect $j_*$ if they were infected by the second epidemic. The process is continued in the obvious fashion to construct the susceptibility set of $i_*$. Note that $i_*$ is infected by the second epidemic if and only if its susceptibility set contains the initial infective.

For large population size *n*, the generation-by-generation growth of the susceptibility set of $i_*$ can be approximated by the corresponding susceptibility set on the coloured tree $\mathcal {T}$, having $i_*$ as its root. The size of $i_*$’s susceptibility set on $\mathcal {T}$ is given by the total progeny of a three-type Galton-Watson process, $\mathcal {B}^{(2)}_B$. As with $\mathcal {B}^{(2)}_F$, the offspring distribution of the initial individual in $\mathcal {B}^{(2)}_B$ is usually different from that of subsequent individuals. For $i \in \{1,3\}$, let $\check{f}_i(\textbf{s})$ be the PGF of the joint offspring distribution of the initial individual in $\mathcal {B}^{(2)}_B$, given that individual has type *i*. For $i \in \{1,2,3\}$, let $\hat{f}_i(\textbf{s})$ be the PGF of the joint offspring distribution of a type-*i* individual in a non-initial generation of $\mathcal {B}^{(2)}_B$. We show in Sect. 6.5 that these PGFs are as follows.

For $i,j \in \{1,2\}$, let (cf. (3.7))

3.16 $$\begin{aligned} \hat{t}_{ij}(s_j)&= 1-\pi _{11}+\pi _{11}s_j,\nonumber \\ \hat{t}_{i3}(s_3)&= 1-\pi _{01}+\pi _{01}s_3,\nonumber \\ \hat{t}_{31}(s_1)&= 1-\pi _{10}+\pi _{10}s_1,\nonumber \\ \hat{t}_{32}(s_2)&= 0,\nonumber \\ \hat{t}_{33}(s_{3})&= 1-\pi _{00}+\pi _{00}s_3. \end{aligned}$$

Then,

3.17 $$\begin{aligned} \hat{f}_{i}(\textbf{s})=f_{\varvec{\tilde{Y}}_i}(\hat{t}_{i1}(s_1),\hat{t}_{i2}(s_2),\hat{t}_{i3}(s_3))\quad (i \in \{1,2,3\}, \textbf{s} \in [0,1]^3), \end{aligned}$$

and

3.18 $$\begin{aligned} \check{f}_{i}(\textbf{s})=f_{\textbf{Y}_i}(\hat{t}_{i1}(s_1),\hat{t}_{i2}(s_2),\hat{t}_{i3}(s_3))\quad (i \in \{1,3\}, \textbf{s} \in [0,1]^3). \end{aligned}$$

In the limit as $n \rightarrow \infty$, in the event of a large second epidemic, the probability that an individual chosen uniformly at random from the population is infected converges to the survival probability of $\mathcal {B}^{(2)}_B$, which leads to the following theorem.

#### Theorem 3.3

Consider the model defined in Sect. 2. Assume that $$R_0^{(2)}>1$$ and that there is a single initial infected individual for the second epidemic. Let $$\hat{\varvec{\xi }}=(\hat{\xi }_{1},\hat{\xi }_{2},\hat{\xi }_{3})$$ be the unique solution in $$[0,1)^{3}$$ of the system of equations

$$\begin{aligned} \hat{\xi }_1&= \hat{f}_1(\hat{\varvec{\xi }}),\\ \hat{\xi }_2&= \hat{f}_2(\hat{\varvec{\xi }}),\\ \hat{\xi }_3&= \hat{f}_3(\hat{\varvec{\xi }}). \end{aligned}$$

For $i \in \{1,3\}$, let $\check{\xi }_i=\check{f}_i(\hat{\varvec{\xi }})$. Then the fraction of vertices infected in a large outbreak is, asymptotically as $n \rightarrow \infty$, given by

$$\begin{aligned} 1-(1-q)\check{\xi }_1-q\check{\xi }_3. \end{aligned}$$

Further, the fraction of vertices infected in the first epidemic that are also infected in the second epidemic is $1-\check{\xi }_1$, and the fraction of vertices not infected in the first epidemic that are infected in the second epidemic is $1-\check{\xi }_3.$

Recall from Sect. 2.2 that the fraction of the population infected by a large first epidemic is $1-q$. Thus, Theorem 3.3 implies that conditional upon both epidemics being large, as $n \rightarrow \infty$, the probability an individual chosen uniformly at random from the population avoids both epidemics converges to $q\check{\xi }_3$, the probability it is infected by the first epidemic but not the second converges to $(1-q)\check{\xi }_1$, the probability it is infected by the second epidemic but not the first converges to $q(1-\check{\xi }_3)$ and the probability it is infected by both epidemics converges to $(1-q)(1-\check{\xi }_1)$.

### Polarized partial immunity

The two models for leaky partial immunity described in Sect. 2.4.2 are special cases of the model for the second epidemic in Sect. 2.3, so Theorems 3.1-3.3 hold for those models, with $\pi _{ij}$ $(i,j \in \{0,1\})$ being given by (2.7) or (2.8) depending on the model. As indicated in Sect. 2.4.1 above, the model for polarized partial immunity described there does not fit the framework of Sect. 2.3. Nevertheless, the proofs of Theorems 3.1–3.3 can be adapted to obtain Theorem 3.4 below.

Recall that under polarized partial immunity, each individual infected by the first epidemic is rendered immune to the second epidemic independently with probability $1-\alpha$. Note that in the construction of $\mathcal {B}^{(2)}_F$, each individual in the tree $\mathcal {T}$ is contacted at most once, i.e., possibly, by its parent in $\mathcal {T}$, so the offspring distributions of $\mathcal {B}^{(2)}_F$ are as in Theorem 3.2, with

3.19 $$\begin{aligned} \pi _{00}= p', \pi _{01}=\alpha p', \pi _{10}=p' \text{ and } \pi _{11}=\alpha p', \end{aligned}$$

where $p'$ is the probability that in the second epidemic an infective infects a given susceptible neighbour who was not infected by the first epidemic, which can be different from *p*, the infection probability in the first epidemic. However, in the construction of $\mathcal {B}^{(2)}_B$, each individual in the tree $\mathcal {T}$ can be contacted several times, i.e. by each of its children in $\mathcal {T}$, so, unless $\alpha =0$ or $\alpha =1$, the offspring distributions of $\mathcal {B}^{(2)}_B$ need modifying accordingly. Note that each red (type-1 or type-2) individual in $\mathcal {T}$ is immune to the second epidemic with probability $1-\alpha$, in which case they can have no offspring in $\mathcal {B}^{(2)}_B$, otherwise they behave the same as a blue (type-3) individual. This is reflected in the forms of the offspring PGFs in Theorem 3.4.

#### Theorem 3.4

Consider the model for polarized partial immunity defined in Sect. 2.4.1 and let $\pi _{ij}$ $(i,j \in \{0,1\})$ be given by (3.19). Then Theorems 3.1 and 3.2 hold for this model. Theorem 3.3 holds also, provided $\hat{f}_{i}(\textbf{s})$ $(i \in \{1,2,3\})$ at (3.17) are replaced by

$$\begin{aligned} \hat{f}_{i}(\textbf{s})=1-\alpha +\alpha f_{\tilde{\textbf{Y}}_i}(1-p'+p's_1,1-p'+p's_2,1-p'+p's_3)\quad (i \in \{1,2\}) \end{aligned}$$

and

$$\begin{aligned} \hat{f}_{3}(\textbf{s})=f_{\tilde{\textbf{Y}}_3}(1-p'+p's_1,1-p'+p's_2,1-p'+p's_3); \end{aligned}$$

and $\check{f}_{i}(\textbf{s})$ $(i \in \{1,3\})$ at (3.18) are replaced by

$$\begin{aligned} \check{f}_1(\textbf{s})=1-\alpha +\alpha f_{\textbf{Y}_1}(1-p'+p's_1,1-p'+p's_2,1-p'+p's_3) \end{aligned}$$

and

$$\begin{aligned} \check{f}_3(\textbf{s})=f_{\textbf{Y}_3}(1-p'+p's_1,1-p'+p's_2,1-p'+p's_3). \end{aligned}$$

## Examples: modelling infection-acquired immunity

In this section, we use our theory to (i) analyse epidemics with polarized and leaky partial immunity caused by the first epidemic that were previously studied through an approximation by Bansal and Meyers (2012); and (ii) investigate herd immunity and the potential of a second wave of an epidemic (cf. Britton et al. (2020)) in a population structured by a configuration model network.

### Polarized partial immunity

In this subsection we investigate the polarized partial immunity model of Sect. 2.4.1 when $p'=p$ (see (3.19)), so apart from some individuals being immune in the second epidemic, the second disease is the same as the first. First, in Sect. 4.1.1, we study how the reproduction number $R_0^{(2)}$ for the second epidemic depends on the probability $\alpha$ that an individual infected by the first epidemic is susceptible to the second epidemic, and determine the critical value of $\alpha$ so that there can be no large second epidemic. Then, in Sect. 4.1.2, we use two concrete examples of networks to explore the accuracy of the approximation of $R_0^{(2)}$ derived by Bansal and Meyers (2012).

#### Critical value of $\alpha$

Consider $R_0^{(2)}$, the largest eigenvalue of the matrix *M* defined at (3.6), with $\pi _{ij}$ $(i,j \in \{0,1\})$ given by (3.19), and use the notation $R_0^{(2)}(\alpha )$ and $M(\alpha )$ to stress the dependence of this reproduction number and offspring mean matrix on $\alpha$. We have trivially that $R_0^{(2)}(1)= R_0^{(1)}$. On the other hand, $R_0^{(2)}(0)<1$, because if $\alpha =0$, the second epidemic spreads among the vertices which were susceptible after the first epidemic and the first epidemic underwent a large outbreak by assumption, so it must be subcritical during its final stage (Newman 2005). Furthermore, from the definition of $R_0^{(2)}(\alpha )$ it is easy to deduce that $R_0^{(2)}(\alpha )$ is a continuous strictly increasing function of $\alpha$ and therefore

$$\begin{aligned} \alpha _{c}=\inf \{\alpha \in [0,1]: R^{(2)}_{0}(\alpha )>1\} \end{aligned}$$

is the unique solution of $R^{(2)}_{0}(\alpha )=1$, which takes its value in (0, 1). Recall that we assume that $R_0^{(1)} >1$.

##### Lemma 1

For polarized partial immunity, $\alpha _c$ is given by the smallest solution in [0, 1] of the quadratic equation

4.1 $$\begin{aligned} &  p^{3}(1-p\mu _{BB})(\mu _{AA}\mu _{BB}-\mu _{AB}\mu _{BA})\alpha ^{2} \nonumber \\ &  \quad -p\left[ (1-p\mu _{BB})(\mu _{AA}+p\mu _{BB})+p(1-p)\mu _{AB}\mu _{BA}\right] \alpha +1-p\mu _{BB}=0.\nonumber \\ \end{aligned}$$

##### Proof

In order to find $\alpha _c$, we note that for polarized partial immunity (with $p'=p$), at least one eigenvalue of $M(\alpha )$ in (3.6) is 1 if and only if $\alpha$ satisfies (4.1). The quadratic equation (4.1) has at least one root in [0, 1], since $\alpha _c\in (0,1)$. If it has two roots in [0, 1], $\alpha _1 <\alpha _2$ say, then $R^{(2)}_0(\alpha _1) \ge 1$ (because $R^{(2)}_0(\alpha _1)$ is the largest eigenvalue of $M(\alpha _1)$, while 1 is an eigenvalue of $M(\alpha _1)$) and $R^{(2)}_0(\alpha _2)> R^{(2)}_0(\alpha _1)$, as $R^{(2)}_0(\alpha )$ is increasing in $\alpha$. Thus, $R^{(2)}_0(\alpha _1)=1$, since otherwise $R^{(2)}_0(\alpha ) \ne 1$ for all $\alpha \in [0,1]$, contradicting $\alpha _c \in (0,1)$. Hence, $\alpha _c$ is the smallest root of (4.1) in [0, 1]. $\square$

#### Example networks

In the first example we consider the regular random graph in which all vertices have degree 3 (i.e. $\mathbb {P}(D=3)=1$). In the second example we assume that for some given $k \in \{2,3,\cdots \}$, $\mathbb {P}(\tilde{D}=1) = \mathbb {P}(\tilde{D}=k)=1/2$. So half of the total degree can be attributed to vertices of degree 1, and the other half to vertices of degree *k*. Furthermore, we assume in this second model that there is no recovery from infection, i.e. $p=1$. For both models we compare the the asymptotically exact threshold parameter $R_0^{(2)}$ given in Theorem 3.4 and the corresponding $\alpha _c$ with the approximations derived by Bansal and Meyers (2012) (see Supplementary Information, Section A). We note that our second example was chosen to highlight that these approximations may differ considerably from the exact results.

In our first example we assume $p\in (1/2,1]$, in order to guarantee that $R_0^{(1)}>1$ (see (2.3)). By using $\mathbb {P}(D=3)=1$ in (3.2)–(3.5) we obtain

$$\begin{aligned} \mu _{AA}=2\left( 1-\frac{(1-p)^{2}}{p}\right) , \quad \mu _{AB}=\frac{2(1-p)^{2}}{p}, \quad \mu _{BA}=\frac{2(2p-1)}{p} \quad \text{ and } \quad \mu _{BB}=\frac{2(1-p)}{p}. \end{aligned}$$

Substituting these values into (4.1) and recalling $p\in (1/2,1]$ yields $\alpha _c$ satisfies $g(\alpha _c)=0$, where

$$\begin{aligned} g(x)= 4p^{3}(1-p)x^{2}-2(1-2p+4p^2-2p^3)x+1. \end{aligned}$$

Note that *g*(*x*) has a minimum, $g(0)=1>0$ and $g(1) =-[p^2(1-p)^2 +(2p-1)^2]<0$. So, in this example, the epidemic threshold $\alpha _{c}$ is the unique solution in [0,1] (and the smallest solution in $[0,\infty )$) of $g(\alpha _c)=0$, which is given by

$$\begin{aligned} \alpha _{c}=\frac{(1-2p+4p^{2}-2p^{3})-\sqrt{(1-2p+4p^{2}-2p^{3})^{2}-4p^{3}(1-p)}}{4p^{3}(1-p)}. \end{aligned}$$

We depict $\alpha _c$ together with the corresponding approximating value from Bansal and Meyers (2012) as a function of *p* in Fig. 2.

**\footnotesize Figure 2:**
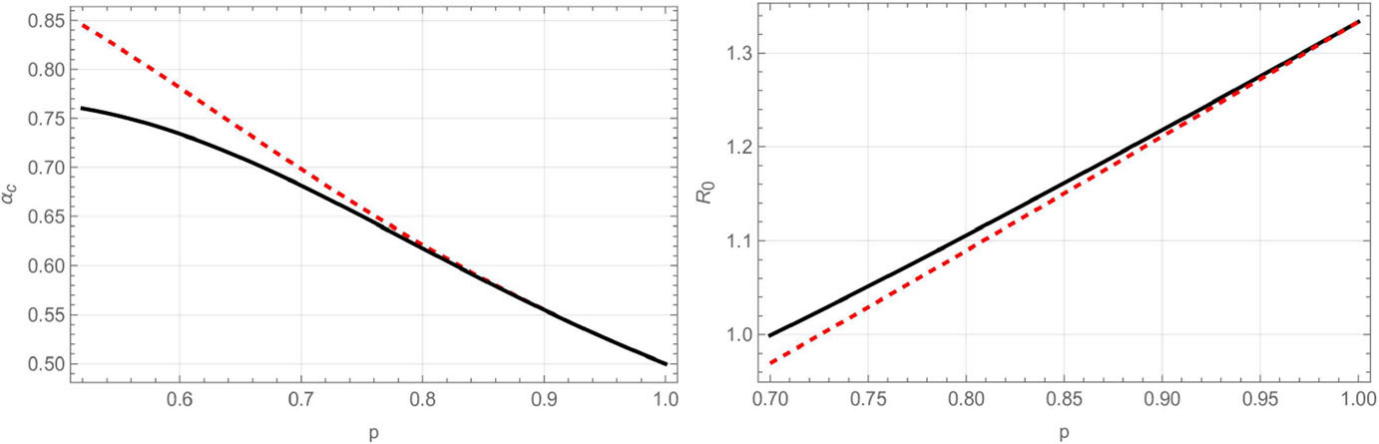
The critical value $\alpha _{c}$ (left) and the basic reproduction number (right) for polarized partial immunity as a function of the transmission probability *p*. In both figures the exact values as deduced in this paper (solid black line) and the approximation of this quantity as given by Bansal and Meyers (2012) (red dashed line) are given for $\mathbb {P}(D=3)=1$. In the right figure $\alpha =2/3$

In Fig. 2 we also plot $R_0^{(2)}(2/3)$ and $R_0^{\text {BM}}(2/3)$ (which is the approximation from Bansal and Meyers (2012) as deduced in equation (A.5) in the Supplementary Information) as functions of *p*. Note that the model by Bansal and Meyers (2012) overestimates $\alpha _{c}$ for this regular network. We also see that, at least on this specific network, the approximation of Bansal and Meyers (2012) is worst for small *p*, but overall offers a reasonable approximation for these parameter values.

In our second example, the constructed graph contains many pairs of neighbours, which have no other neighbours, while if *k* is large most of the other vertices are in one giant component. Note that pairs of neighbours of degree 1 play no role in the spread of a large epidemic, whether it is the first or second epidemic. Recall that $p=1$, so for large *k*, $\tilde{q} \approx 1/2$ and $R_0^{(2)}(\alpha ) \approx (k-1) \alpha /2$. In Fig. 3 we compare $R_0^{(2)}(\alpha )$ with $R_0^{\text {BM}}(\alpha )$. We see in this example (which is constructed exactly for this purpose), that the difference between the exact $R_0^{(2)}(\alpha )$ and the approximation $R_0^{\text {BM}}(\alpha )$ may be considerable. In particular, the approximation of Bansal and Meyers (2012) yields $\alpha _c \approx 0.38$, while in fact the true value is approximately $2/(k-1) =0.2$.

The intuition behind the approximation failing in this particular case, is that Bansal and Meyers (2012) implicitly assume that the network is “rewired” among just the “survivors” from the first epidemic before the second epidemic starts. Since vertices of degree 1 always end paths of infection, the rewiring means that some vertices of degree 1 that were neighbours of each other before rewiring now block long paths in the network on which the second epidemic spreads, which leads to a lower reproduction number for the second epidemic.

**\footnotesize Figure 3:**
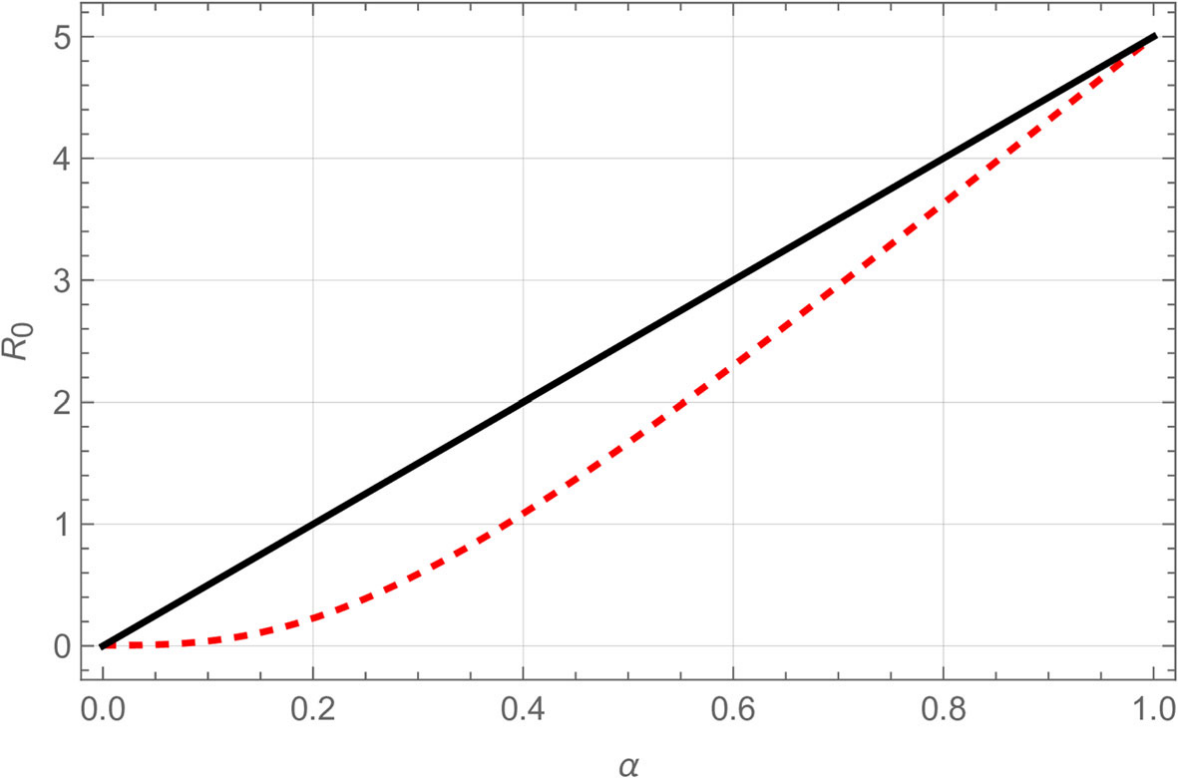
The basic reproduction number for polarized partial immunity as a function of $\alpha$, the fraction of individuals infected in the first epidemic that is susceptible again for the second epidemic, as deduced in this paper (solid black line) and the approximation of this quantity as given in Bansal and Meyers (2012) (red dashed line). Here $\mathbb {P}(\tilde{D}=1)=\mathbb {P}(\tilde{D}=11)=1/2$ and $p=1$

### Leaky partial immunity

In this subsection we consider similar examples to those in Sect. 4.1, using the model for leaky partial immunity obtained by setting $p'=p$ in (2.7). Using (3.6), the offspring mean matrix for the second epidemic is

4.2 $$\begin{aligned} M_{\textrm{leaky}}=p\begin{pmatrix} \alpha _S \alpha _I\mu _{AA} & p \alpha _S \alpha _I \mu _{AB} & (1-p)\alpha _I\mu _{AB} \\ \alpha _S \alpha _I \mu _{BA} & p \alpha _S \alpha _I \mu _{BB} & (1-p)\alpha _I\mu _{BB} \\ \alpha _S\mu _{BA} & 0 & \mu _{BB} \end{pmatrix}. \end{aligned}$$

The corresponding mean matrix under the model for polarized partial immunity defined in Sect. 2.4.1, with $p'=p$, is

4.3 $$\begin{aligned} M_{\textrm{pol}}=p\begin{pmatrix} \alpha \mu _{AA} & p \alpha \mu _{AB} & (1-p)\mu _{AB} \\ \alpha \mu _{BA} & p \alpha \mu _{BB} & (1-p) \mu _{BB} \\ \alpha \mu _{BA} & 0 & \mu _{BB} \end{pmatrix}. \end{aligned}$$

Suppose that $\alpha =\alpha _S \alpha _I$ and $\alpha _I>0$, and let $\Delta$ be the $3 \times 3$ diagonal matrix with successive diagonal elements $1, 1, \alpha _I^{-1}$. Then $M_{\textrm{leaky}}=\Delta M_{\textrm{pol}} \Delta ^{-1}$, so $M_{\textrm{leaky}}$ and $M_{\textrm{pol}}$ have the same eigenvalues. It follows that the basic reproduction number $R_0^{(2)}$ for the second epidemic is the same under both the leaky and polarized models for partial immunity, and hence so is the critical value $\alpha _c$.

For computations, Bansal and Meyers (2012) again use an approximation in which they “reconfigure” the edges in the configuration model between the first and second epidemic. This reconfiguring is performed in such a way that whether or not a vertex is infected in the first epidemic, as well as its number of infected neighbours in the first epidemic are not changed. Bansal and Meyers (2012) analyse the second epidemic (on the reconfigured network) using a two-type bond percolation model. Although this approach yields an analysis that is exact in the large population limit for an epidemic on the reconfigured network, it does not lead to an asymptotically exact analysis of the second epidemic as the reconfigured network does not yield an asymptotically correct model for the true network following the first epidemic. The approximating two-type bond percolation model is described in Supplementary Information, Section B, where the corresponding value of $\alpha$, which we denote by $\alpha _c^{\textrm{BM}}$, so that the second epidemic is critical is derived. As shown in Section B of the Supplementary Information, the joint-degree distributions given in Bansal and Meyers (2012) that underpin their analysis of the two-type bond percolation model are incorrect. The correct distributions are used in the examples below. This second approximation in Bansal and Meyers (2012) can also be used for polarized partial immunity and under it $R_0^{(2)}$, and hence also $\alpha _c^{\textrm{BM}}$, is the same for both leaky and polarized partial immunity when $\alpha _S \alpha _I =\alpha$ (see Section B of the Supplementary Information).

**\footnotesize Figure 4:**
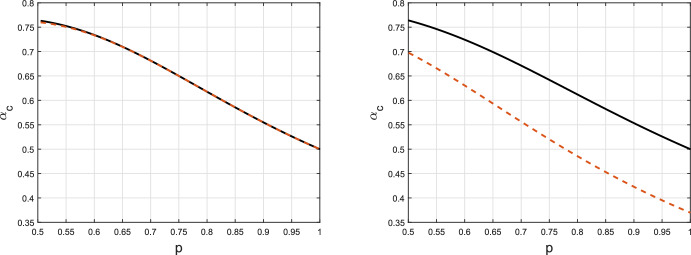
The critical value $\alpha _c$ as a function of the transmission probability *p* as deduced in this paper (solid black line) and the approximation of this quantity using the two-type percolation approximation (dashed red line). Left panel: $\mathbb {P}(D=3)=1$; right panel: $\mathbb {P}(\tilde{D}=1)=\mathbb {P}(\tilde{D}=5)=1/2$. See text for further details

We depict $\alpha _c$ and $\alpha _c^{\textrm{BM}}$ as functions of *p* for the degree distributions given by (i) $\mathbb {P}(D=3)=1$ and (ii) $\mathbb {P}(\tilde{D}=1)=\mathbb {P}(\tilde{D}=5)=1/2$ in Fig. 4. Note that both of these degree distributions have $\mathbb {E}[\tilde{D}-1]=2$ and hence $R_0^{(1)}=2p$. For the first degree distribution, the approximation $\alpha _c^{\textrm{BM}}$ is extremely good, indeed for polarized partial immunity it is clearly superior to that used in Fig. 2. However, the approximation performs poorly for the second degree distribution. In particular, when $p=1$, the approximation $\alpha _c^{\textrm{BM}}$ is appreciably smaller than the correct $\alpha _c$, which has the following intuitive explanation. When $p=1$, after the first epidemic there is no edge between an infected and a susceptible individual and in the event of a large outbreak all individuals of the giant component of the underlying network are infected. Thus the reconfiguration of edges between the first and second epidemic underlying the approximation simply involves breaking all edges between individuals infected by the first epidemic into half-edges and repairing them uniformly at random, and doing the same for edges between individuals not infected by the first epidemic. The degree distribution of individuals in the giant component is stochastically larger than *D*, so the approximation leads to an inflated $R_0^{(2)}$, and hence a reduced $\alpha _c$.

### The herd immunity threshold after a first outbreak

When an epidemic spreads through a population, often measures are taken to limit the impact of the disease. If a vaccine is available this can be done through vaccinating (a part of) the population. If a vaccine is not available, non-pharmaceutical interventions have to be taken in order to limit the number of contacts of individuals and/or the probability that a contact leads to transmission of the epidemic.

To incorporate the above in our model, we first assume that infection confers complete immunity, so $\pi _{11}= \pi _{01}=0$ and our model reduces to that of Newman (2005). The second epidemic involves only individuals who were not infected by the first epidemic, and its early stages are approximated by a single-type branching process, whose offspring mean is $R_0^{(2)} =\pi _{00} \mu _{BB}$ (cf. the bottom-right element of the matrix *M* defined in (3.6)). Thus, using (3.5),

4.4 $$\begin{aligned} R_0^{(2)}=\pi _{00} \sum _{k=2}^{\infty }(k-1) \tilde{p}_k (1-p+p\tilde{q})^{k-2} = \pi _{00} \mathbb {E}[(\tilde{D}-1) (1-p+p\tilde{q})^{\tilde{D}-2}],\nonumber \\ \end{aligned}$$

where $\tilde{q}$ is the smallest positive solution of (2.5).

Now assume that $\pi _{00}$ is the probability that an infectious individual makes an infectious contact with a given neighbour in an unrestricted epidemic and

$$\begin{aligned} R_0'=\pi _{00} \mathbb {E}[\tilde{D}-1] >1, \end{aligned}$$

so if there was no large first epidemic, an unrestricted epidemic is supercritical. We investigate the threshold value of *p*, the infection probability for the first epidemic, for which herd immunity is obtained, that is, the value of *p* for which $R_0^{(2)}=1$ holds. We then compute the corresponding value of $1-q$, the fraction of the population that is no longer susceptible. In a homogeneously mixing population the latter value is $1-1/R^{\text {hom}}_0$, where $R^{\text {hom}}_0$ is the basic reproduction number for the unrestricted epidemic in a homogeneously mixing population (Diekmann et al. (2012), p. 69).

Note that $R_0^{(2)}$ is non-increasing in *p* (because increasing *p* leaves fewer individuals susceptible after the first outbreak). Further, for $p \mathbb {E}[\tilde{D}-1]>1$, it follows from (2.5) that $\tilde{q}$ is strictly decreasing and continuous in *p* and hence, using (4.4), so is $R_0^{(2)}$. Let $p'$ be chosen such that $R_0^{(2)} =1$, and let $\tilde{q}'$ and $q'$ be the corresponding $\tilde{q}$ and *q*, respectively. Then we obtain

4.5 $$\begin{aligned} 1 =\pi _{00} \mathbb {E}[(\tilde{D}-1) (1-p'+p'\tilde{q}')^{\tilde{D}-2}] = \pi _{00} \frac{ \mathbb {E}[D(D-1) (1-p'+p'\tilde{q}')^{D-2}]}{\mathbb {E}[D]}\nonumber \\ \end{aligned}$$

and, using (2.4),

$$\begin{aligned} q' =\mathbb {E}[(1-p'+p'\tilde{q}')^{D}]. \end{aligned}$$

Further, note that

$$\begin{aligned} R'_0= \pi _{00} \mathbb {E}[\tilde{D}-1] = \pi _{00} \frac{\mathbb {E}[D(D-1)]}{\mathbb {E}[D]}. \end{aligned}$$

In general it is not possible to give a closed-form expression for $q'$ in terms of “standard functions”. However, suppose that *D* has a Poisson-type distribution, i.e. its PGF satisfies $f'_D(s) = f_D'(1) \left( f_D(s)\right) ^{\kappa }$ for some $\kappa \in (0,\infty )$ (Jacobsen et al. 2018). Then, $f_{\tilde{D}-1}(s)=\left( f_D(s)\right) ^{\kappa }$ and $f_{\tilde{D}-1}'(s)=\kappa \left( f_D(s)\right) ^{2\kappa -1}\mu _D$. Further, it follows from (2.4) and (2.5) that $\tilde{q}=q^{\kappa }$, so $f_D(1-p+p\tilde{q})=q^{1/\kappa }$. Hence, $R'_0=\pi _{00}\kappa \mu _D$ and the first equality in (4.5) yields after a little algebra that

4.6 $$\begin{aligned} q'=(1/R_0')^{1/(2\kappa -1)}, \end{aligned}$$

so we get an explicit expression for the disease-induced herd immunity level. Moreover, note that $1-q' < 1-1/R_0'$ if and only if $\kappa >1$. Thus, the herd immunity threshold obtained through letting a less infectious disease spread through the population is strictly less than the herd immunity level obtained through immunisation of a uniformly chosen subset of the vertices of an appropriate size if and only if $\kappa >1$ (cf. Britton et al. (2020)).

Some examples of Poisson-type distributions are (i) $D \sim \textrm{Bin}(n, \theta )$, in which case $\kappa = (n-1)/n$, so $q'=(1/R_0')^{n/(n-2)}$, (ii) $D \sim \textrm{NegBin}( r, \theta )$ (with support $\mathbb {Z}_{\ge 0}$), in which case $\kappa = (r+1)/r$, so $q'=(1/R_0')^{r/(r+2)}$, (iii) $D \sim \textrm{Poisson}(\lambda )$, in which case $\kappa =1$, so $q'=1/R_0'$. Note that $\mathbb {P}(D=n)=1$ for some integer *n*, is a special case of $D \sim \textrm{Bin}(n, \theta )$, with $\theta =1$.

Returning to the general case, observe that, $1-q' \le 1-1/R_0'$ if and only if

4.7 $$\begin{aligned} \mathbb {E}[D(D-1) (1-p'+p'\tilde{q}')^{D-2}]\le \mathbb {E}[D(D-1)]\mathbb {E}[(1-p'+p'\tilde{q}')^{D}].\end{aligned}$$

Further, if *D* has a Mixed-Poisson distribution with mean distribution *X*, i.e.

$$\begin{aligned} \mathbb {P}(D=k)=\mathbb {E}[\textrm{e}^{-X}X^k]/k! \quad (k=0,1,\dots ) \end{aligned}$$

for some non-negative random variable *X*, then (4.7) reads

$$\begin{aligned} \mathbb {E}[X^2 \textrm{e}^{-p'(1-\tilde{q}')X}] \le \mathbb {E}[X^2] \mathbb {E}[\textrm{e}^{-p'(1-\tilde{q}')X}], \end{aligned}$$

which is always the case by Chebychev’s other inequality (Hardy et al. (1952), p.168), because $x^2$ is increasing for $x >0$ and $\textrm{e}^{-p'(1-\tilde{q}')x }$ is decreasing for $x>0$. Note that the Poisson distribution is a special case of a Mixed-Poisson distribution.

## Numerical illustrations of our model

In this section, we use simulations to explore the accuracy for finite networks of our asymptotic results for the probability ($p_{\textrm{maj}}$) and relative final size ($z_{\textrm{maj}}$) of a large second epidemic, and investigate briefly the behaviour of $z_{\textrm{maj}}$ on the type (polarized or leaky) of partial immunity and other model parameters, especially the choice of degree distribution. The examples are based on (Bansal and Meyers (2012), Section 3). We consider four degree distributions:

(i) $D \equiv d$, i.e. *D* is constant with $p_d=1$ and $f_D(s)=s^d$;
(ii) $D \sim \textrm{Poisson}(\mu _D)$, i.e. *D* is Poisson with mean $\mu _D$ and $f_D(s)=\textrm{e}^{-\mu _D(1-s)}$;
(iii) $D \sim \textrm{Geom}(\theta )$, i.e. $p_k=(1-\theta )^{k-1} \theta$ $(k=1,2,\dots )$, with mean $\mu _D=\theta ^{-1}$ and $f_D(s)=\theta s/(1-(1-\theta )s$);
(iv) $D \sim \textrm{Power}(\alpha _D,\kappa )$, i.e. $p_k=ck^{-\alpha _D}\textrm{e}^{-\frac{k}{\kappa }}$ $(k=1,2,\dots )$, where $\alpha _D, \kappa \in (0,\infty )$ and the normalising constant $c=\textrm{Li}_{\alpha _D}(\textrm{e}^{-\frac{1}{\kappa }})$, with $\textrm{Li}_{\alpha _D}$ being the polylogarithm function.

### Remark 5.1

The fourth distribution is a power law with exponential cut-off (see, for example, Newman (2002)) that has been used extensively in the physics literature. Note that, with $\theta =\textrm{e}^{-\frac{1}{\kappa }}$ and $\beta =\textrm{Li}_{\alpha _D}(\theta )$, $f_D(s)=\beta ^{-1}\textrm{Li}_{\alpha _D}(\theta s)$, $f_D^{(1)}(s)=\frac{1}{\beta s}\textrm{Li}_{\alpha _D-1}(\theta s)$ and $f_D^{(2)}(s)=\frac{1}{\beta s^2}[\textrm{Li}_{\alpha _D-2}(\theta s)- \textrm{Li}_{\alpha _D-1}(\theta s)]$, which enable $R_0^{(1)}, R_0^{(2)}, p_{\textrm{maj}}$ and $z_\textrm{maj}$ to be calculated. Bansal and Meyers (2012) use the scale-free distribution $p_k=k^{-\gamma }/\zeta (\gamma )$ $(k=1,2,\dots )$, where $\zeta$ is the Riemann zeta function and $\gamma$ $(\approx 2.0654)$ is chosen so that $\mu _D=10$. However, that distribution has infinite variance, which violates the assumptions in Sect. 2.1.

### Relating asymptotic theory to outcomes in finite populations

Each simulation for a population of size *n* consists of first simulating $D_1, D_2, \dots , D_n$ from the given degree distribution, yielding the numbers of half-edges attached to the individuals in the population, and then pairing these half-edges uniformly at random to form the underlying network, as described in Sect. 2.1. Two successive epidemics are then simulated on the network so constructed. For the first epidemic, the initial infective is chosen uniformly at random from the population. For the second epidemic, under polarized partial immunity, each individual infected in the first epidemic is rendered immune independently with probability $1-\alpha$, and the initial infective is then chosen uniformly at random from all individuals that remain susceptible. Under leaky partial immunity, an individual, $i_*$ say, is chosen uniformly at random from the whole population. If $i_*$ was not infected by the first epidemic then it is the initial infective for the second epidemic. If $i_*$ was infected by the first epidemic then with probability $\alpha _S$ it is the initial infective for the second epidemic, otherwise a new potential initial infective $i_*'$ is chosen independently according to the same law, and the process is repeated until an initial infective is obtained.

Let $p^I_1$ and $p^I_2$ be the individual-to-individual infection probabilities for the two epidemics, assuming no immunity in the second epidemic. We set $p^I_1=0.15, p^I_2=0.3$ and consider networks with $\mu _D=10$, as in (Bansal and Meyers (2012), Fig. 5). The values of $\sigma _D^2$ under the four distributions are (i) 0, (ii) 10, (iii) 90 and (iv) 271.4950, yielding values of $R_0^{(1)}$, calculated from (2.3), of 1.35, 1.5, 2.7 and 5.4224, respectively. We use the formulation of leaky partial immunity given by (2.7) with $p'=p^I_2$, so $R_0^{(2)}$ is the same under the two models of partial immunity provided $\alpha _S \alpha _I=\alpha$. In particular, the two $R_0^{(2)}$s are equal if $(\alpha _S, \alpha _I)=(\sqrt{\alpha }, \sqrt{\alpha )}$ and that is used, with $\alpha =\frac{1}{2}$, in Table 1, which gives estimates of the probability, $p_{\textrm{maj}}^{(n)}$, and relative final size, $z_{\textrm{maj}}^{(n)}$, of a large second epidemic, conditional upon a large first epidemic, for a network of size *n*, obtained from simulations as follows.

For each combination of population size *n*, degree distribution and partial immunity type, 10, 000 simulations of a network and two successive epidemics on it were performed. The estimate $\hat{p}_{\textrm{maj}}^{(n)}$ of $p_{\textrm{maj}}^{(n)}$ is given by the fraction of those simulations that had a large first epidemic that also had a large second epidemic, while the estimate $\hat{z}_{\textrm{maj}}^{(n)}$ of $z_{\textrm{maj}}$ is given by the sample mean of the fractions of the population infected in the latter second epidemics. Standard errors of the estimates are given in brackets in Table 1. An epidemic in a population having $n=500$ was deemed large if its size exceeded 100. If $n=1,000$ the cut-off was 200, whilst for $n=5,000$ and $n=10,000$ the cut-off was 300. These cut-offs were chosen by inspection of corresponding histograms. For the parameter values used in the simulations there is a clear distinction between small and large epidemics. The asymptotic values $p_{\textrm{maj}}$ and $z_{\textrm{maj}}$, calculated using Theorems 3.2–3.4 in Sect. 3, are given in the $n=\infty$ rows of the table. The method of choosing the initial infective for the second epidemic implies that the probability that infective is of type 3 is $q/(q+\alpha (1-q))$ for polarized partial immunity and $q/(q+\alpha _S(1-q))$ for leaky partial immunity.

**\bf Table 1:** Simulation results against theoretical (asymptotic) calculations for probability and relative final size of a large second epidemic, given a large first epidemic

*D*	*n*	Polarized		Leaky	
		$\hat{p}_{\textrm{maj}}^{(n)}$	$\hat{z}_{\textrm{maj}}^{(n)}$	$\hat{p}_{\textrm{maj}}^{(n)}$	$\hat{z}_{\textrm{maj}}^{(n)}$
	500	0.8705 (0.0046)	0.6369 (0.0009)	0.8382 (0.0050)	0.8389 (0.0006)
	1000	0.8720 (0.0046)	0.6333 (0.0006)	0.8457 (0.0049)	0.8393 (0.0004)
$D \equiv 10$	5000	0.8680 (0.0046)	0.6313 (0.0003)	0.8403 (0.0049)	0.8399 (0.0002)
	10000	0.8704 (0.0045)	0.6315 (0.0002)	0.8359 (0.0050)	0.8400 (0.0001)
	$\infty$	0.8720	0.6311	0.8508	0.8397
	500	0.8018 (0.0053)	0.5764 (0.0008)	0.7969 (0.0053)	0.7984 (0.0005)
	1000	0.8040 (0.0052)	0.5743 (0.0005)	0.7935 (0.0053)	0.7984 (0.0004)
$\text{ Poisson }(10)$	5000	0.8111 (0.0051)	0.5741 (0.0002)	0.7973 (0.0053)	0.7984 (0.0002)
	10000	0.7990 (0.0052)	0.5740 (0.0002)	0.7930 (0.0053)	0.7986 (0.0001)
	$\infty$	0.8098	0.5738	0.8053	0.7986
	500	0.5591 (0.0066)	0.4127 (0.0007)	0.6437 (0.0064)	0.6481 (0.0005)
	1000	0.5738 (0.0066)	0.4114 (0.0005)	0.6475 (0.0064)	0.6472 (0.0003)
$\text{ Geom }(1/10)$	5000	0.5686 (0.0066)	0.4110 (0.0002)	0.6553 (0.0064)	0.6469 (0.0001)
	10000	0.5725 (0.0066)	0.4106 (0.0002)	0.6438 (0.0064)	0.6467 (0.0001)
	$\infty$	0.5706	0.4106	0.6294	0.6468
	500	0.4242 (0.0073)	0.3294 (0.0008)	0.5258 (0.0074)	0.5310 (0.0005)
	1000	0.4242 (0.0073)	0.3292 (0.0006)	0.5216 (0.0073)	0.5309 (0.0004)
$\text{ Power }(1,36.6472)$	5000	0.4225 (0.0073)	0.3275 (0.0003)	0.5313 (0.0073)	0.5305 (0.0002)
	10000	0.4443 (0.0073)	0.3274 (0.0002)	0.5221 (0.0074)	0.5305 (0.0001)
	$\infty$	0.4273	0.3274	0.5037	0.5304

Table 1 indicates that for these parameter values the asymptotic approximations of both $p_{\textrm{maj}}$ and $z_{\textrm{maj}}$ are good, even for $n=500$, with the approximation of $\hat{z}_{\textrm{maj}}^{(n)}$ being generally better than that of $\hat{p}_{\textrm{maj}}^{(n)}$. Note that under this calibration, polarized partial immunity gives better protection in the sense that the size of a large second epidemic, if one occurs, is smaller. However, for the constant and Poisson degree distributions, the probability of a large second epidemic is slightly larger. Note also that for degree distributions in Table 1, the probability and mean size of a large second epidemic both decrease as $\sigma _D^2$ increases. This is because the first epidemic is greater for larger $\sigma _D^2$ (cf. the values of $R_0^{(1)}$ given above) and hence the population is better protected against the second epidemic.

### Exploring model behaviour

Figure 5 shows plots of the fraction of the population infected by a large second epidemic, $z_{\textrm{maj}}$, against the individual-to-individual infection probability for the second epidemic, $p_2^I$, for various models of partial immunity when the individual-to-individual infection probability for the first epidemic, $p_1^I=0.15$. For polarized partial immunity, we set $\alpha =\frac{1}{2}$, as before. For leaky partial immunity, we consider three models with $\alpha _S\alpha _I=\frac{1}{2}$ and also the model with $\alpha _S=\alpha _I=\frac{1}{2}$. The latter uses the calibration considered by Bansal and Meyers (2012), who note that “the total susceptibility and total transmissibility over the entire network is equal in the two immunity models". However, for disease transmission these factors tend to operate in a multiplicative rather than additive fashion. If $\alpha _S=\alpha _I=\alpha$, then every element of $M_{\textrm{leaky}}$ (see (4.2)) is less than or equal to the corresponding element of $M_{\textrm{pol}}$ (see (4.3)), with strict inequality for some elements provided $\alpha \in (0,1)$. It follows, in an obvious notation, that if $\alpha \in (0,1)$ then $R_{0,\text {leaky}}^{(2)} <R_{0,\text {pol}}^{(2)}$, as reflected in the intercepts of the curves in Fig. 5 with the $p_2^I$-axis. If instead $\alpha _S=\alpha _I=\sqrt{\alpha }$, then $R_{0,\text {leaky}}^{(2)} = R_{0,\text {pol}}^{(2)}$, as is also reflected by the intercepts of the curves in Fig. 5.

**\footnotesize Figure 5:**
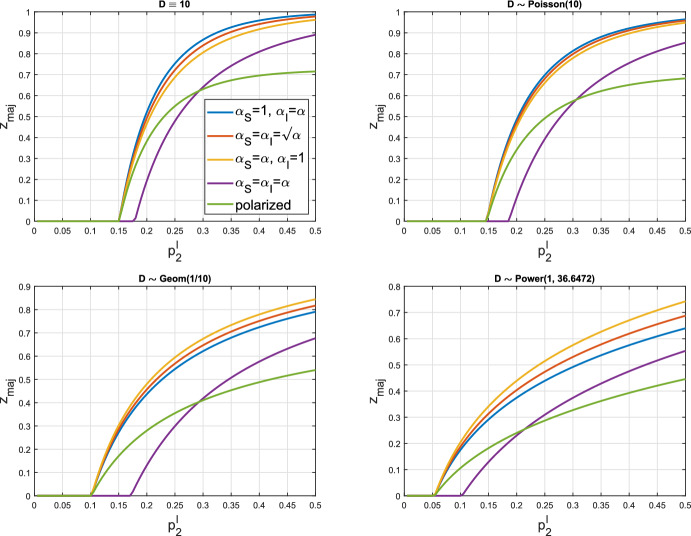
Relative final size $z_{\textrm{maj}}$ of a large second outbreak. See text for further details

One way of considering the effects of the two models of partial immunity is to imagine that each individual infected by the first epidemic is given two coins, a susceptibility coin that has probability $\alpha _S$ of landing heads and an infectivity coin that has probability $\alpha _I$ of landing heads. Under the leaky model, every time a susceptible individual is contacted they toss their susceptibility coin and become infected if and only if it lands heads. If they become infected then they attempt to contact their neighbours independently, each with probability $p_2^I$, but for each such attempt they toss their infectivity coin and the contact is successful if and only if it lands heads. Under the polarized model, the first time that a susceptible is contacted they toss their susceptibility coin. If it lands heads then they become infected and then contact their neighbours independently, each with probability $p_2^I$; they do not toss their infectivity coin. However, if it lands tails then they are immune from that contact and all subsequent contacts.

Suppose that $(\alpha _S, \alpha _I)=(\alpha ,1)$. Then it is easily seen that polarized partial immunity (with parameter $\alpha$) leads to a smaller second epidemic than leaky partial immunity, since an individual’s susceptibility coin landing tails protects them also from *all* further contacts. Suppose instead that $(\alpha _S, \alpha _I)=(\alpha ,\alpha )$. Then leaky partial immunity is likely to lead to a smaller second epidemic at low values of $p_2^I$, since individuals are unlikely to be contacted twice in the second epidemic and individuals infected in both the first and second epidemics are less infectious than under polarized partial immunity. However, at high values of $p_2^I$, individuals are likely to be contacted several times in the second epidemic and the advantage gained from the lifelong immunity in the polarized model more than offsets individuals being more infectious if infected. This explains the crossover effect of the corresponding curves in Fig. 5.

## Analysis of the second epidemic and proofs

### A percolation approach to the first and second epidemic

To prove Theorems 3.1 and 3.2, we use, as sketched in Sect. 2.3, a colouring of the vertices and edges in the graph $\mathcal {G}$ and in a tree $\mathcal {T}$, in which the “local neighbourhood” of vertices is distributed as the local neighbourhood of vertices in $\mathcal {G}$ as $n \rightarrow \infty$. In this section the basic reproduction number of the first epidemic $R_0^{(1)}$, as defined in (2.3), and the random variable describing the asymptotic degree distribution *D* and its size-biased variant $\tilde{D}$, as defined in Sect. 2.1, play key roles. Throughout this section we assume that $R_0^{(1)}>1$ and that a large first epidemic has occurred.

#### Colouring the graph $\mathcal {G}$

In this and the following subsection we give a justification of the coloured tree approach given in Sect. 2.3. In what follows we exploit that in the first epidemic, for different pairs of neighbours, the events that the individual first infected (if any) makes an infectious contact with the other are independent and all have probability *p*. We do this through colouring the vertices and edges in $\mathcal {G}$. The coloured graph we denote by $\mathcal {G}_*^{(n)} =\mathcal {G}_*= (\mathcal {V}_*,\mathcal {E}_*)$, and we now describe its construction.

Note that each edge in $\mathcal {E}$ can be used for transmission of the disease at most once during an epidemic, namely, when one of the endpoints is infectious and the other susceptible, since after such a contact both endpoints of the edge are infectious (Cox and Durrett 1988). Hence, for every edge in $\mathcal {G}$, we may determine in advance whether or not it serves as a possible route of transmission of infection, provided one of its end vertices becomes infected. We create $\mathcal {E}_*$ by colouring the edges in $\mathcal {E}$ independently, each edge being red with probability *p* and blue otherwise. By construction, the subset of red edges in $\mathcal {E}_*$ is distributed as the set of open edges after bond percolation with edge-probability *p* on the graph $\mathcal {G}$ (Cox and Durrett 1988; Grimmett 1999). Red edges are interpreted as possible routes of transmission of the disease, that is, if there is a red edge present between two vertices, then the first vertex that becomes infectious (if any) infects the second vertex, if the second vertex has not been infected by another vertex in the meantime.

Let $v_0$ denote the initially infectious vertex in the first epidemic. We obtain $\mathcal {V}_*$ by colouring the vertices in $\mathcal {V}$ such that $v_0$ and all vertices in $\mathcal {V}$ which can be reached by a red path in $\mathcal {E}_*$ from $v_0$ are coloured red, while all other vertices are blue. The set of red vertices is denoted by $\mathcal {C}(v_0)$. By construction, the set of individuals infected during the first epidemic is distributed as $\mathcal {C}(v_0)$. So, loosely speaking, the red vertices and edges are those that are infected and along which infectious contacts would be made if one of the end-vertices of the edge got infected during the first epidemic.

Using the obvious connection between $\mathcal {G}_*$ and percolation on $\mathcal {G}$ we call a subset $\mathcal {S}$ of $\mathcal {V}_*$ a component if $\mathcal {S}$ is connected through red edges in $\mathcal {G}_*$ (i.e. every vertex in $\mathcal {S}$ can be reached from all other vertices in $\mathcal {S}$ through a path of red edges in $\mathcal {E}_*$) and there is no red edge connecting $\mathcal {S}$ to its complement. If $R_0^{(1)}>1$ we say that the epidemic or outbreak is large if $\mathcal {C}(v_0)$ is the largest component of $\mathcal {V}_*$. We denote this largest component by $\mathcal {C}_{\infty }$. With probability tending to 1 as $n \rightarrow \infty$, $\mathcal {C}_{\infty }$ is the only component which contains more than $(\log n)^2$ vertices and there exists a constant $q <1$, defined in (2.4), such that the fraction of vertices in the largest component converges in probability to $1-q$ as $n \rightarrow \infty$ (Durrett (2007), Thm. 3.2.2). It follows immediately that in the first epidemic the probability of a large outbreak converges to $1-q$ and conditioned on a large outbreak the fraction of the population infected converges in probability to $1-q$.

#### A coloured tree approximation of $\mathcal {G}_*$

For the second epidemic we assume that the first outbreak is large and therefore that $\mathcal {C}(v_0) = \mathcal {C}_{\infty }$. We assume implicitly that $R_0^{(1)}>1$. We consider the coloured graph $\mathcal {G}_*$ and pick a vertex of $\mathcal {G}_*$, say $w_0$, uniformly at random. This vertex represents the initial case for the second epidemic. Then $w_0$ may or may not be part of the cluster $\mathcal {C}_{\infty }$. By construction of the graph $\mathcal {G}$, the environment of $w_0$ is with high probability locally tree-like, i.e. for any $k \in \mathbb {N}$ the number of circuits (a circuit is a path of *distinct* edges for which the starting vertex of the first edge is the same as the end vertex of the last edge) in $\mathcal {G}$ that both contain $w_0$ and are of length at most *k* converges in probability to 0 as $n \rightarrow \infty$. We use this locally tree-like property of $\mathcal {G}$ to describe the environment of $w_0$ first in $\mathcal {G}_*$ and then in $\mathcal {G}$. We call $w_0$ the root of the tree.

Let $\mathcal {N}^{(n)}(w_0;k)$ be the set of (coloured) vertices within distance *k* of $w_0$ in $\mathcal {G}_*^{(n)}$, together with the (coloured) edges with both end-vertices in this set. Let $\mathcal {T}$ be a graph created by a two-stage Galton-Watson tree (van der Hofstad (2016), Chapter 3) in which the ancestor (root) has offspring distribution *D* and the other particles in the Galton-Watson tree have offspring distribution $\tilde{D}-1$, i.e. including their parent they have degree distribution $\tilde{D}$. The vertex set of $\mathcal {T}$ corresponds to the particles in the Galton-Watson process and vertices are connected by an edge if and only if they have a parent–child relationship. Let $\mathcal {T}_*$ be a coloured version of $\mathcal {T}$, in which edges are independently red with probability *p*, and otherwise blue. A vertex is coloured red if it is part of an infinite path of red edges in $\mathcal {T}_*$. Formally this means that a vertex at distance *d* ($d \in \mathbb {N}$) from the root is red if and only if it is connected through a path of red edges to at least one vertex at every distance larger than *d* from the root ($w_0$). Note that if $\mathcal {T}$ is finite (which occurs with strictly positive probability if $p_0+p_1>0$), then all vertices in $\mathcal {T}_*$ are blue. Furthermore, note that we allow for the infinite red path from a red vertex at distance *d* of the root, to go via its neighboor at distance $d-1$ of the root. Let $\mathcal {T}_*(k)$ be the subgraph of $\mathcal {T}_*$ consisting of all vertices within distance *k* of the root and the edges of $\mathcal {T}$ with both end-vertices in this set, while the colour of vertices and edges is preserved.

We use without formal proof that for all $k \in \mathbb {N}$, $\mathcal {N}^{(n)}(w_0;k)$ converges in distribution to $\mathcal {T}_*(k)$ as $n \rightarrow \infty$. See Newman (2002) for a heuristic discussion of the convergence of the non-coloured version of the graphs and Ball et al. (2009) for a more formal analysis. The convergence of the coloured graphs can be understood heuristically by observing that with high probability, i.e. with probability tending to 1 as $n \rightarrow \infty$, the unique large component in $\mathcal {G}_*$ corresponds to the infinite components in $\mathcal {T}_*$. Note that in large but finite graphs, two uniformly chosen vertices that are part of large components are with high probability in the same component, although their distance goes to infinity as $n \rightarrow \infty$ (Durrett (2007), p. 84), while on a percolated infinite tree, different vertices may be in different infinite components (which can be interpreted as the vertices being at infinite distance from each other).

We then analyse the second epidemic as if it spreads on $\mathcal {T}_*$ starting with the root initially infectious. We assume that the infectivity and susceptibility of the red vertices behave in the same way as they do in $\mathcal {G}_*$. We use, again without a formal proof, that the probability of a large second outbreak on $\mathcal {G}_*$ converges to the probability of an infinite outbreak on $\mathcal {T}_*$, and we define and compute a natural threshold parameter $R_0^{(2)}$ for the second epidemic. In the remainder of the paper we say that for every non-root vertex *u* in $\mathcal {T}_*$, the neighbour of *u* in $\mathcal {T}_*$ which is on the (self-avoiding) path from the root to *u* is the *parent* of *u*. All other neighbours are *children* of *u*. Similarly, if *u* is on the path from the root to a vertex *v*, then *v* is a *descendant* of *u*. Finally, we call an infinite path in $\mathcal {T}_*$ of descendants of *u* which starts at *u* a *path of descent of u*. We say that a path of descent is red if and only if all edges in the path are red.

The spread of the first epidemic creates three types of vertices: vertices which were infected in the first epidemic and have a red path of descent (type 1), vertices which were infected in the first epidemic but have no red path of descent (type 2), and vertices which escaped the first epidemic and therefore have no red path of descent (type 3). Note that vertices of type 2 are always connected by a red edge to their parent which is either of type 1 or of type 2. Note also that a vertex *u* in $\mathcal {T}_*$ is of type 1 if and only if a red path of descent in $\mathcal {T}_*$ starts at *u*. This typing of vertices is necessary to construct a branching process approximation to the second epidemic. Below we see that the joint distributions of the numbers of children of the various types depends on the type of the individual/vertex whose children are under consideration, and therefore the different types are needed.

In Fig. 1 in Sect. 2.3, we provide an illustration of $\mathcal {T}$ with $p_3 = \mathbb {P}(D=3) =1$. The vertices $u_7$, $u_{17}$ and $u_{19}$ are of type 2. All other red vertices are of type 1, and all blue vertices are of type 3.

The typing of the vertices in $\mathcal {T}_*$ allows us to approximate the second epidemic on $\mathcal {T}_*$ by a multi-type Galton-Watson process (Jagers 1975, Chapter 4) in which the ancestor has, in general, a different offspring distribution than all other individuals in the process. (It follows easily from (3.1) and the uniqueness of PGFs that *D* and $\tilde{D}-1$ have the same distribution if and only if $D \sim \text {Poisson}(\mu _D)$ for some $\mu _D$.) We denote this branching process by $\mathcal {B}_F^{(2)}$. The superfix (2) indicates that the approximation is for the second epidemic. The suffix *F* indicates that the branching process approximates the forward process of the epidemic, in contrast to the branching process $\mathcal {B}_B^{(2)}$ (see Sect. 6.5), which approximates the backward process (or susceptibility set (Ball 2019)).

### Branching process approximation for the second epidemic

For $i,j \in \{1,2,3\}$, let $\tilde{X}_{ij}$ be the random number of type-*j* vertices infected by a type-*i* vertex in $\mathcal {T}_*$, which is not the root. Define $m_{i j}= E[\tilde{X}_{ij}]$ and *M* as the $3 \times 3$ matrix with $m_{ij}$ as the *i*, *j* element. That is, *M* is the mean offspring matrix for the non-initial generations of $\mathcal {B}_F^{(2)}$. Note that this matrix is the same as *M* defined in (3.6). The dominant eigenvalue of the matrix *M* is a threshold parameter for the second epidemic and is denoted by $R^{(2)}_{0}$. A major outbreak of the second epidemic occurs asymptotically with strictly positive probability if and only if $R^{(2)}_{0}>1$.

To make further progress we introduce two labels of vertices, *A* and *B*. The vertices with label *A* are the vertices with a red path of descent in $\mathcal {T}_*$, while the vertices with label *B* do not have a red path of descent. So, we label type-1 vertices by *A*, and type-2 and type-3 vertices by *B*. The number of children of a typical (i.e. non-root) vertex in $\mathcal {T}$ is distributed as $\bar{D}-1$ and a vertex has label *B*, if each of its children either has label *B* or is connected to that vertex by a blue edge. Therefore, the probability that a vertex in the tree has label *B* satisfies (2.5) and standard theory from branching processes gives that it is the smallest positive solution of this equation. That is, the probability that a typical vertex in the tree has label *B* is $\tilde{q}$, while the probability that a vertex in the tree has label *A* is $1-\tilde{q}$. We note that by Bayes’ Theorem, for integers $0\le \ell \le k-1$, the probability that a typical vertex (say *v*) with label *A* has $k-1$ children in total (i.e. *v* is of degree *k*), of which $\ell$ are of type *A*, satisfies

6.1 $$\begin{aligned} \mathbb {P}&( v \text { has }k-1 \text { children of which }\ell \text { have label }A \mid v \text { has label } A )\nonumber \\&\qquad =\mathbb {P}(v \text { has } k-1 \text { children of which } \ell \text { have label } A)\nonumber \\&\qquad \qquad \times \frac{\mathbb {P}(v \text { has label }A \mid v \text { has } k-1 \text { children of which } \ell \text { have label } A)}{\mathbb {P}(v \text { has label} A)}. \end{aligned}$$

Because *v* has label *A* if and only if *v* shares a red edge with at least one child with label *A*, we have

$$\begin{aligned} \mathbb {P}(v \text { has label } A \mid v \text { has }k-1 \text { children of which }\ell \text { have label } A) = 1-(1-p)^{\ell } \end{aligned}$$

and (6.1) yields

6.2 $$\begin{aligned} \mathbb {P}&( v \text { has } k-1 \text { children of which }l \,\,\text { have label }A \mid v\text { has label }A )\nonumber \\&\qquad = \tilde{p}_k \genfrac(){0.0pt}0{k-1}{\ell }(1-\tilde{q})^{\ell } \tilde{q}^{k-\ell -1} \times \frac{ 1-(1-p)^{\ell }}{1-\tilde{q}}. \end{aligned}$$

We can compute in a similar fashion,

6.3 $$\begin{aligned} \mathbb {P}&( v \text { has } k-1 \text { children of which }\ell \text { have label }A \mid v \text { has label }B )\nonumber \\&\qquad = \tilde{p}_k \genfrac(){0.0pt}0{k-1}{\ell }(1-\tilde{q})^{\ell } \tilde{q}^{k-\ell -1} \times \frac{ (1-p)^{\ell }}{\tilde{q}}. \end{aligned}$$

Obviously, because all children have either label *A* or *B*, we can deduce

$$\begin{aligned} \mathbb {P}( v \text { has } k-1 \text { children of which }\ell \text { have label }B \mid v \text { has label }i ), \end{aligned}$$

for $i \in \{A,B\}$, immediately from (6.2) and (6.3). Below we use the above probabilities to compute the threshold parameter $R_0^{(2)}$, and the probability and size of a large outbreak of the second epidemic.

### Proof of Theorem 3.1: computation of $R_0^{(2)}$

In order to compute $R_0^{(2)}$, which is the dominant eigenvalue of the mean offspring matrix *M*, we first need to derive, for $i,j \in \{A,B\}$, the expected number of label-*j* children of a given label-*i* individual, which we denote by $\mu _{ij}$. Expressions for these expectations are derived as follows, using (6.2) and (6.3). Let *v* be a vertex, which is not the root. By definition we have

$$\begin{aligned} \mu _{AA}&=\mathbb {E}[\text { number of label-}A \text { children of } v \mid v\,\, \text {has label }\, A ]\\&=\frac{ \mathbb {E}\left[ (\text { number of label-}A \text { children of }v)\,\mathbbm {1} (v \text { has label }A)\right] }{\mathbb {P}(v \text { has label}\, A)}\\&=\frac{\sum _{\ell =1}^{\infty }\ell \,\mathbb {P}(v \text { has } \ell \text { label-}\, A \,\,\text {children})\,\mathbb {P} (v \text { has label } A \mid v \text { has }\ell \,\,\text {label-A children})}{\mathbb {P}(v\,\, \text {has label}\, A)}. \end{aligned}$$

Conditioning on the degree of *v* then gives

$$\begin{aligned} \mu _{AA}&= \frac{1}{1-\tilde{q}} \displaystyle \sum _{k=1}^{\infty }\tilde{p}_{k}\displaystyle \sum _{\ell =0}^{k-1} \ell \genfrac(){0.0pt}0{k-1}{\ell }(1-\tilde{q})^{\ell } \tilde{q}^{k-\ell -1}(1-(1-p)^{\ell })\\&=\displaystyle \sum _{k=2}^{\infty }(k-1)\tilde{p}_{k}\displaystyle \sum _{\ell =1}^{k-1}\genfrac(){0.0pt}0{k-2}{\ell -1}(1-\tilde{q})^{l-1} \tilde{q}^{k-\ell -1}(1-(1-p)^{\ell }). \end{aligned}$$

Finally, using the binomial theorem, we obtain (3.2):

$$\begin{aligned} \mu _{AA} =\displaystyle \sum _{k=2}^{\infty }(k-1)\tilde{p}_{k}\left( 1-(1-p)(1-p+p\tilde{q})^{k-2}\right) . \end{aligned}$$

Note that $\mu _{AA}$ may be written as

$$\begin{aligned} \frac{d}{dx} \left. \displaystyle \sum _{k=2}^{\infty }\tilde{p}_{k}x^{k-1}\right| _{x=1} - (1-p) \frac{d}{dx} \left. \displaystyle \sum _{k=2}^{\infty }\tilde{p}_{k} x^{k-1}\right| _{x=1-p+p\tilde{q}}, \end{aligned}$$

so

$$\begin{aligned} \mu _{AA}= &  f_{\tilde{D}-1}'(1) -(1-p)f_{\tilde{D}-1}'(1-p+p \tilde{q})\\= &  \frac{1}{\mu _D} \left( f_D''(1) -(1-p)f_D''(1-p+p \tilde{q})\right) . \end{aligned}$$

(Recall that $f_{\tilde{D}-1}(x)=\mathbb {E}[x^{\tilde{D}-1}]$ and $f_D(x)=\mathbb {E}[x^D]$ ($x \in [0,1]$) are the PGFs for the random variables $\tilde{D}-1$ and *D*, respectively.) Similarly, we obtain (3.3),

$$\begin{aligned} \mu _{AB}&=\frac{1}{1-\tilde{q}} \displaystyle \sum _{k=1}^{\infty }\tilde{p}_{k}\displaystyle \sum _{\ell =0}^{k-1}\genfrac(){0.0pt}0{k-1}{\ell }(1-\tilde{q})^{\ell } \tilde{q}^{k-\ell -1}(k-\ell -1)(1-(1-p)^{\ell })\\&=\frac{\tilde{q}}{1-\tilde{q}} \displaystyle \sum _{k=2}^{\infty }(k-1)\tilde{p}_{k}\displaystyle \sum _{\ell =0}^{k-2}\genfrac(){0.0pt}0{k-2}{\ell }(1-\tilde{q})^{\ell } \tilde{q}^{k-\ell -2}(1-(1-p)^{\ell })\\&=\frac{\tilde{q}}{1-\tilde{q}}\displaystyle \sum _{k=2}^{\infty }(k-1)\tilde{p}_{k}\left( 1-(1-p+p\tilde{q})^{k-2}\right) , \end{aligned}$$

so

$$\begin{aligned} \mu _{AB}= \frac{1}{\mu _D} \left( f_D''(1) -f_D''(1-p+p \tilde{q})\right) . \end{aligned}$$

With similar computations we obtain (3.4) and (3.5), where we can write

$$\begin{aligned} \mu _{BA}&= \frac{(1-p)(1-\tilde{q})}{\tilde{q}}\displaystyle \sum _{k=2}^{\infty }(k-1)\tilde{p}_{k}(1-p+p\tilde{q})^{k-2}\\&= \frac{(1-p)(1-\tilde{q})}{\tilde{q}} \frac{1}{\mu _D} f_D''(1-p+p \tilde{q}) \end{aligned}$$

and

$$\begin{aligned} \mu _{BB}= \displaystyle \sum _{k=2}^{\infty }(k-1)\tilde{p}_{k}(1-p+p\tilde{q})^{k-2}= \frac{1}{\mu _D} f_D''(1-p+p \tilde{q}). \end{aligned}$$

As noted in Sect. 3.1, a label-*B* child of a red individual is type 2 if and only if it is infected by its parent in the first epidemic, and all label-*B* children of a blue individual are necessarily type 3. So we obtain the matrix M from (3.6):

$$\begin{aligned} M=\begin{pmatrix} \pi _{11}\mu _{AA} & p\,\pi _{11}\mu _{AB} & (1-p)\pi _{10}\mu _{AB} \\ \pi _{11}\mu _{BA} & p\,\pi _{11}\mu _{BB} & (1-p)\pi _{10}\mu _{BB} \\ \pi _{01}\mu _{BA} & 0 & \pi _{00}\mu _{BB} \end{pmatrix}. \end{aligned}$$

The threshold parameter $R^{(2)}_0$ is given by the dominant eigenvalue of the matrix *M* (Diekmann et al. 2012, Theorem 7.3) and Theorem 3.1 follows (see also (Jagers (1975), Chapter 4)).

### Proof of Theorem 3.2: the probability of a large outbreak

In order to prove Theorem 3.2 and compute the probability of a large outbreak of the second epidemic, we need to compute the PGFs of the joint offspring distributions of $\mathcal {B}_F^{(2)}$. We need to consider the offspring random variables for the root of the branching process (as defined at the start of Sect. 6.1.2), and the non-initial generations of the approximating branching process separately.

Note that the root can only be of type 1 or type 3. For $i \in \{1,3\}$, let $\textbf{Y}_i=(Y_{i1},Y_{i2},Y_{i3})$ be a random vector, where $Y_{ij}$ ($j \in \{1,2,3\}$) is the number of type-*j* children of the root of $\mathcal {T}_*$ if this root is of type *i*, and let $\textbf{X}_i=(X_{i1},X_{i2},X_{i3})$ denote a random vector, where $X_{ij}$ ($j \in \{1,2,3\}$) is the number of type-*j* children infected in the second epidemic by the root of $\mathcal {T}_*$ if this root is of type *i*. To understand the difference between $Y_{ij}$ and $X_{ij}$ note that whether or not vertex *v* is a child of the root does not depend on the spread of the second epidemic, but only on whether or not *v* is a neighbour of the root of $\mathcal {T}_*$.

For $\textbf{s}=(s_{1},s_{2},s_{3})\in [0,1]^3$, define the PGFs

$$\begin{aligned} f_1(\textbf{s})= &  \mathbb {E}[s_{1}^{X_{11} }s_{2}^{X_{12} } s_{3}^{X_{13}}],\\ f_3(\textbf{s})= &  \mathbb {E}[s_{1}^{X_{31}} s_{3}^{X_{33} }],\\ f_{\textbf{Y}_1}(\textbf{s})= &  \mathbb {E}[s_{1}^{Y_{11} }s_{2}^{Y_{12} } s_{3}^{Y_{13}}],\\ f_{\textbf{Y}_3}(\textbf{s})= &  \mathbb {E}[s_{1}^{Y_{31} }s_{2}^{Y_{32} } s_{3}^{Y_{33}}]. \end{aligned}$$

We show that these definitions are consistent with (3.8). Observe that $f_3(\textbf{s}) = \mathbb {E}[s_{1}^{X_{31}} s_{2}^{X_{32}} s_{3}^{X_{33} }]$, because $X_{32}=0$ by definition. To compute $f_{i}(\textbf{s})$ ($i\in \{1,3\}$), we use that

6.4 $$\begin{aligned} f_{i}(\textbf{s})=\mathbb {E}[s_{1}^{X_{i1} }s_{2}^{X_{i2} } s_{3}^{X_{i3} }] = \mathbb {E}\left[ \mathbb {E}\left[ s_{1}^{X_{i1} }s_{2}^{X_{i2} } s_{3}^{X_{i3} }\mid Y_{i1},Y_{i2},Y_{i3}\right] \right] . \end{aligned}$$

Because infections in the second epidemic are independent, we have

6.5 $$\begin{aligned} &  \mathbb {E}[s_{1}^{X_{11} }s_{2}^{X_{12} } s_{3}^{X_{13} }|Y_{11},Y_{12},Y_{13}]]\nonumber \\ &  \quad =(1-\pi _{11}+\pi _{11}s_1)^{Y_{11}}(1-\pi _{11}+\pi _{11}s_2)^{Y_{12}}(1-\pi _{10}+\pi _{10}s_3)^{Y_{13}}. \end{aligned}$$

Similarly,

6.6 $$\begin{aligned} \mathbb {E}[s_{1}^{X_{31} }s_{2}^{X_{32} } s_{3}^{X_{33} }|Y_{31},Y_{32},Y_{33}]] = (1-\pi _{01}+\pi _{01}s_1)^{Y_{31}}(1-\pi _{00}+\pi _{00}s_3)^{Y_{33}},\nonumber \\ \end{aligned}$$

where we have used that $X_{32} =0$.

Recall from (3.7) that, for $i,j \in \{1, 2\},$

$$\begin{aligned} t_{ij}(s_j)= &  1-\pi _{11}+\pi _{11}s_j,\\ t_{i3}(s_3)= &  1-\pi _{10}+\pi _{10}s_3,\\ t_{31}(s_1)= &  1-\pi _{01}+\pi _{01}s_1,\\ t_{32}(s_2)= &  0,\\ t_{33}(s_{3})= &  1-\pi _{00}+\pi _{00}s_3. \end{aligned}$$

It immedialtely follows from (6.4)-(6.6) that, for $i\in \{1,3\}$ and $\textbf{s}=(s_{1},s_{2},s_{3})\in [0,1]^3$,

$$\begin{aligned} f_{i}(\textbf{s}) = f_{\textbf{Y}_i}(t_{i1}(s_1),t_{i2}(s_2),t_{i3}(s_3)). \end{aligned}$$

To complete the derivation of $f_{i}(\textbf{s})$ $(i \in \{1,3\})$ we determine $f_{\textbf{Y}_i}(\textbf{s})$ $(i \in \{1,3\})$. First,

6.7 $$\begin{aligned} f_{\textbf{Y}_1}(\textbf{s})&=\frac{1}{1-q}\displaystyle \sum _{k=1}^{\infty } p_{k}\displaystyle \sum _{\ell _{1}=0}^{k}\genfrac(){0.0pt}0{k}{\ell _{1}}(1-\tilde{q})^{\ell _{1}} \tilde{q}^{k-\ell _{1}}(1-(1-p)^{\ell _1})\nonumber \\&\qquad \times \displaystyle \sum _{\ell _{2}=0}^{k-\ell _1}\genfrac(){0.0pt}0{k-\ell _1}{\ell _{2}}p^{\ell _{2}} (1-p)^{k-\ell _{1}-\ell _2} s_1^{\ell _1}s_2^{\ell _2}s_3^{k-\ell _1-\ell _2}\nonumber \\&=\frac{1}{1-q}\displaystyle \sum _{k=1}^{\infty }p_{k}\big (\left( 1-\tilde{q})s_1+\tilde{q}(p s_2+(1-p)s_3\right) \big )^{k}\nonumber \\&\qquad -\frac{1}{1-q}\displaystyle \sum _{k=1}^{\infty }p_{k}\big (\left( 1-p)(1-\tilde{q})s_1+\tilde{q}(ps_2+ (1-p)s_3\right) \big )^{k}\nonumber \\&=\frac{1}{1-q}f_{D}(\left( 1-\tilde{q})s_1+\tilde{q}(p s_2+(1-p)s_3\right) )\nonumber \\&\qquad -\frac{1}{1-q}f_{D}(\left( 1-p)(1-\tilde{q})s_1+\tilde{q}(p s_2+(1-p)s_3\right) ). \end{aligned}$$

The first equality can be understood by noting that if a vertex (*v* say) of degree *k* has an (infinite) red path of descent at least one of *v*’s children has to have a red path of descent, and if there are $\ell _1$ children with a red path of descent, at least one of them has to be connected through a red edge to *v*, hence the $1-(1-p)^{\ell _1}$ term. The remaining $k-\ell _1$ children do not have a red path of descent and among those $k-\ell _1$, $\textrm{Bin}(k-\ell _{1},p)$ are of type 2 (cf. the derivation of (6.2)). The second equality follows using the binomial theorem.

In the same way we deduce

6.8 $$\begin{aligned} f_{\textbf{Y}_3}(\textbf{s}) = \frac{1}{q}\displaystyle f_{D}\left( (1-p)(1-\tilde{q})s_1+\tilde{q}s_3\right) . \end{aligned}$$

Next we consider the offspring in $\mathcal {B}^{(2)}_F$ of individuals other than the root. For $i,j \in \{1,2,3\}$, let $\tilde{\textbf{Y}}_i=(\tilde{Y}_{i1},\tilde{Y}_{i2},\tilde{Y}_{i3})$ denote a random vector where $\tilde{Y}_{ij}$ is the number of type-*j* children of a type-*i* individual in $\mathcal {T}_*$ that is not the root. Moreover, for $i,j \in \{1,2,3\}$, let $\tilde{\textbf{X}}_i=(\tilde{X}_{i1},\tilde{X}_{i2},\tilde{X}_{i3})$ be a random vector, where $\tilde{X}_{ij}$ is the number of type-*j* vertices infected in the second epidemic by a type-*i* vertex in $\mathcal {T}_*$, which is not the root. For $\textbf{s}=(s_{1},s_{2},s_{3})\in [0,1]^3$ and $i \in \{1,2,3\}$, let $f_{\tilde{\textbf{Y}}_i}(\textbf{s})=\mathbb {E}[s_{1}^{\tilde{Y}_{i1} }s_{2}^{\tilde{Y}_{i2} } s_{3}^{\tilde{Y}_{i3} }]$.

Using the same arguments as above we obtain that

6.9 $$\begin{aligned} \tilde{f}_{i}(\textbf{s})=f_{\tilde{\textbf{Y}}_i}(t_{i1}(s_1),t_{i2}(s_2),t_{i3}(s_3))\quad (i \in \{1,2,3\}, \textbf{s} \in [0,1]^3). \end{aligned}$$

Furthermore, by similar calculations to the derivations of (3.9) and (3.10), we obtain (3.12)–(3.14).

The extinction probability (and hence the probability of a minor outbreak) is now computed using the theory of multi-type Galton-Watson processes (Jagers (1975), Chapter 4). Assume that $R_0^{(2)}>1$.

Let $\varvec{\xi }=(\xi _{1},\xi _{2},\xi _{3})$ be the unique solution of the system of equations

$$\begin{aligned} \xi _1 = \tilde{f}_1(\xi _1,\xi _2,\xi _3), \quad \xi _2 = \tilde{f}_2(\xi _1,\xi _2,\xi _3) \quad \text{ and } \quad \xi _3 = \tilde{f}_3(\xi _1,\xi _2,\xi _3) \end{aligned}$$

in $$[0,1)^{3}$$. Then for $$i \in \{1,2,3\}$$, $$\xi _i$$ is the probability that the (second epidemic) offspring of an infected type-*i* individual (other than the root) goes extinct. Thus, the probability of a minor outbreak if the root of $$\mathcal {G}_*$$ is of type *i* ($$i \in \{1,3\}$$) is given by $$f_{i}(\varvec{\xi })$$. The unconditional probabilities of minor and large outbreaks depend on how the root is chosen. If the root is a vertex chosen uniformly from the population, then the probability of a large outbreak is $$1-(1-q) f_{1}(\varvec{\xi })-q f_{3}(\varvec{\xi })$$. This finishes the proof of Theorem 3.2.

We note that it might be more natural to let the probability that a vertex is the root of the second epidemic depend on the susceptibility of this vertex, in which case the computation of the probability of the root is dependent on the specific model under consideration (see Sect. 5).

### Proof of Theorem 3.3: final size of a large outbreak

A key tool in determining the fraction of the population that is infected by a large outbreak, in the limit as the population size $n \rightarrow \infty$ is the concept of *susceptibility sets* (see e.g. Ball and Neal (2002); Ball (2019); Britton et al. (2019)). To introduce this concept, consider the first epidemic and relax the assumption that the infectious period random variable *L* is almost surely constant.

Construct a random directed graph, which we denote by $\mathcal {G}_1$, on the vertex set $\mathcal {V}$ as follows. For each $i \in \mathcal {V}$, determine who *i* would contact if they were to become infected by first sampling its infectious period $L_i$ independently from *L* and then, conditional upon $L_i$, drawing a directed edge from *i* to each of its neighbours in the graph independently with probability $1-\textrm{e}^{-\beta L_i}$.

For distinct *i* and *j* in $\mathcal {V}$, write $i \leadsto j$ if and only if there is a chain of directed edges from *i* to *j*. For $i \in \mathcal {V}$, define the susceptibility set of *i* by $\mathcal {S}_i^{(1)}=\{j \in \mathcal {V} \setminus \{i\}: j \leadsto i\}$. Note that an initially susceptible individual, *i* say, is infected by the first epidemic if and only if $\mathcal {S}_i^{(1)}$ has non-empty intersection with the set of initial infectious individuals, hence the terminology.

Suppose that *n* is large and that the epidemic is started by a single initial infectious individual chosen uniformly at random from $\mathcal {V}$. The size $S_i^{(1)}$ of the susceptibility set $\mathcal {S}_i^{(1)}$ can be approximated by the total progeny of a *backward* Galton-Watson branching process, $\mathcal {B}_B^{(1)}$ say, defined as follows. The branching process $\mathcal {B}_B^{(1)}$ has one ancestor, who corresponds to individual *i* and is excluded from the above total progeny. Note that *i* has degree distributed according to *D* and each of *i*’s neighbours would infect *i* independently with probability $p=1-\mathbb {E}[\textrm{e}^{-\beta L}]$ if they were to become infected. Thus the offspring random variable for the initial generation of $\mathcal {B}_B^{(1)}$ has the mixed-binomial distribution $\textrm{Bin}(D,p)$, that is a binomial distribution with random “size parameter” *D* and “probability parameter” *p*. Now consider a typical neighbour, *j* say, of *i* that joins the susceptibility set $\mathcal {S}_i$. The number of neighbours of *j* is distributed according to $\tilde{D}$ but one of those neighbours is *i*, so the offspring random variable for this and all subsequent generations of $\mathcal {B}_B^{(1)}$ has the mixed-binomial distribution $\textrm{Bin}(\tilde{D}-1,p)$. The generation-based approximation of the susceptibility set $\mathcal {S}_i^{(1)}$ by the branching process $\mathcal {B}_B^{(1)}$ continues in the obvious fashion.

It is intuitively plausible that for large *n*, individual *i* is infected by a large outbreak if and only if the branching process $\mathcal {B}_B^{(1)}$ that approximates $S_i$ does not go extinct. Further, let $Z_n^{(1)}$ denote the size of the first epidemic. Then, subject to mild regularity conditions, conditional upon the occurrence of a large outbreak (more formally one that infects at least $\log n$ individuals), $n^{-1}Z_n^{(1)}$ converges in probability to $1-q$ as $n \rightarrow \infty$, where *q* is the extinction probability of $\mathcal {B}_B^{(1)}$; see Ball et al. (2014) for a proof when the underlying graph $\mathcal {G}$ is a random intersection graph. It is easily checked that *q* is given by (2.4).

The approach with susceptibility sets is close to a probability generating functions approach to calculate the size of the giant in-component in directed or semi-directed configuration model networks as described in (Kenah and Robins 2007b; Miller 2007; Kenah and Miller 2011). The susceptibility set of a node is then identical to the in-component of the node in the corresponding epidemic percolation network. For the first epidemic, neither the calculations using susceptibility sets nor those using epidemic percolation networks become conceptually harder, when not assuming fixed infectious periods (Ball and Neal 2002; Kenah and Robins 2007a; Ball et al. 2009). However, as discussed in Sect. 7, the analysis of the second epidemic becomes harder if one does not assume a fixed infectious period for the first epidemic.

We now assume that for the first epidemic the infectious period is fixed and has length 1 (i.e. $\mathbb {P}(L=1)=1$) and consider the second epidemic, assuming that the first epidemic resulted in a large outbreak. Let $\mathcal {G}_2$ denote the random directed graph corresponding to $\mathcal {G}_1$ above but for the second epidemic; i.e. in which there is a directed edge from *i* to *j* if and only if individual *i*, if infected by the second epidemic, contacts individual *j* in that epidemic. As in Sect. 6.1.2, pick a root $w_0$ uniformly at random from all vertices in $\mathcal {V}$, independently from the root chosen in Sect. 6.1.2. The susceptibility set, $\mathcal {S}_{w_0}^{(2)}$ say, of individual $w_0$ for the second epidemic is derived from $\mathcal {G}_2$ in the obvious fashion. The susceptibility set $\mathcal {S}_{w_0}^{(2)}$ can be approximated using a three-type Galton-Watson process, $\mathcal {B}_B^{(2)}$ say, with individuals typed as in the “forward process” of infection analysed in Sect. 6.1.2, but with the new choice of $w_0$. As in Sect. 6.4, the root $w_0$ is necessarily of type 1 or 3.

For $i,j \in \{1,2,3\}$, let $\hat{X}_{ij}$ be the number of type-*j* offspring of a typical type-*i* individual in a non-initial generation of the branching process $\mathcal {B}_B^{(2)}$ and define $\check{X}_{ij}$ $(i \in \{1,3\}, j \in \{1,2,3\})$ similarly for the initial generation. Further, for $\textbf{s} \in [0,1]^3$, let

$$\begin{aligned} \begin{array}{rl} \hat{f}_{i}(\textbf{s}) & =\mathbb {E}[s_{1}^{\hat{X}_{i1} }s_{2}^{\hat{X}_{i2} } s_{3}^{\hat{X}_{i3} }] \quad (i \in \{1,2,3\}),\\ \check{f}_{i}(\textbf{s}) & =\mathbb {E}[s_{1}^{\check{X}_{i1} }s_{2}^{\check{X}_{i2} } s_{3}^{\check{X}_{i3} }] \quad (i \in \{1,3\}). \end{array} \end{aligned}$$

For $$i \in \{1,3\}$$, let $$\check{\xi }_i$$ be the extinction probability of $$\mathcal {B}_B^{(2)}$$ given that there is one ancestor, whose type is *i*. Omitting the details, it is easily verified that if $$R_0^{(2)}>1$$ then $$\check{\xi }_1<1$$ and $$\check{\xi }_3<1$$, otherwise $$\check{\xi }_1=\check{\xi }_3=1$$. By standard multi-type branching process theory (cf. the end of Sect. 6.4), if $$R_0^{(2)}>1$$ then $$\check{\xi }_i=\check{f}_i(\hat{\varvec{\xi }})$$, $$i \in \{1,3\}$$, where $$\hat{\varvec{\xi }}=(\hat{\xi }_1, \hat{\xi }_2, \hat{\xi }_3)$$ is the unique solution in $$[0,1)^{3}$$ of the system of equations $$\hat{\xi }_i = \hat{f}_i(\hat{\varvec{\xi }})$$, $$i \in \{1,2,3\}$$.

Let $Z_n^{(2)}$ be the size of the second epidemic. Note that, in the limit as $n \rightarrow \infty$, given a large first epidemic, the root $w_0$ has type 1 with probability $1-q$ (where *q* is given by (2.4)) otherwise it has type 3. Then, given that the first and second epidemics are both large, we conjecture that $n^{-1}Z_n^{(2)}$ converges in probability to $z=1-(1-q)\check{\xi }_1-q\check{\xi }_3$ as $n \rightarrow \infty$. (This conjecture is supported by simulations, see Sect. 5, and also by equivalent results for other models; for example (Ball et al. (2009), Thm 6.2)).

To compute $\check{\xi }_1$ and $\check{\xi }_3$, we need expressions for the PGFs $\hat{f}_{i}(\textbf{s})$ $(i \in \{1,2,3\})$ and $\check{f}_{i}(\textbf{s})$ $(i \in \{1,3\})$. We use that

6.10 $$\begin{aligned} \hat{f}_{i}(\textbf{s}) =\mathbb {E}[\mathbb {E}[s_{1}^{\hat{X}_{i1} }s_{2}^{\hat{X}_{i2} } s_{3}^{\hat{X}_{i3} }|\tilde{Y}_{i1},\tilde{Y}_{i2},\tilde{Y}_{i3}]] \quad (i \in \{1,2,3\}) \end{aligned}$$

and

6.11 $$\begin{aligned} \check{f}_{i}(\textbf{s}) =\mathbb {E}[\mathbb {E}[s_{1}^{\check{X}_{i1} }s_{2}^{\check{X}_{i2} } s_{3}^{\check{X}_{i3} }| Y_{i1},Y_{i2},Y_{i3}]] \quad (i \in \{1,3\}), \end{aligned}$$

where $\tilde{Y}_{ij}$ $(i,j \in \{1,2,3\})$ and $Y_{ij}$ $(i \in \{1,3\}, j \in \{1,2,3\})$ are as defined in Sect. 6.4.

The calculations of $\mathbb {E}[s_{1}^{\hat{X}_{i1} }s_{2}^{\hat{X}_{i2} } s_{3}^{\hat{X}_{i3} }|\tilde{Y}_{i1},\tilde{Y}_{i2},\tilde{Y}_{i3}]$ for $i \in \{1,2,3\}$ and $\mathbb {E}[s_{1}^{\check{X}_{i1} }s_{2}^{\check{X}_{i2} } s_{3}^{\check{X}_{i3} }|Y_{i1},Y_{i2},Y_{i3}]$ for $i \in \{1,3\}$ are essentially the same as those for the corresponding conditional PGFs used in determining the probability of a large outbreak in Sect. 6.4, except that $\pi _{ij}$ $(i,j \in \{0,1\})$ defined at (2.6) are replaced by $\hat{\pi }_{ij}$ $(i,j \in \{0,1\})$, where $\hat{\pi }_{ij}=\pi _{ji}$ $(i,j \in \{0,1\})$. This is because the branching process $\mathcal {B}_B^{(2)}$ approximates the backward process associated with the second epidemic, whereas $\mathcal {B}_F^{(2)}$ approximates the corresponding forward process. It follows using (6.10) that

$$\begin{aligned} \hat{f}_{i}(\textbf{s})=f_{\tilde{\textbf{Y}}_i}(\hat{t}_{i1}(s_1), \hat{t}_{i2}(s_2), \hat{t}_{i3}(s_3))\quad (i \in \{1,2,3\}, \textbf{s} \in [0,1]^3), \end{aligned}$$

where $\hat{t}_{ij}(s_j)$ $(i,j \in \{1,2,3\})$ are given by (3.16). Similarly,

$$\begin{aligned} \check{f}_1(\textbf{s})=f_{\textbf{Y}_i}(\hat{t}_{i1}(s_1),\hat{t}_{i2}(s_2),\hat{t}_{i3}(s_3))\quad (i \in \{1,3\}, \textbf{s} \in [0,1]^3). \end{aligned}$$

The expressions for $\hat{f}_{i}(\textbf{s})$ $(i \in \{1,2,3\})$ and $\check{f}_1(\textbf{s})$ $(i \in \{1,3\})$ coincide with those given at (3.17) and (3.18), so Theorem 3.3 follows.

### Proof of Theorem 3.4: polarized partial immunity

We analyse the model with polarized partial immunity by considering the spread of the second epidemic on the coloured tree $\mathcal {T}_*$ (see Sect. 6.1.2) in a similar fashion as is done above for the model of Sect. 2.3. For the analysis of the probability of a large outbreak of an epidemic on $\mathcal {T}_*$ (or on any tree) it is important that an individual can only be infected by its parent. So, whether or not a vertex is infected by its parent in $\mathcal {T}_*$ is independent of other infection events in the tree. Therefore, we may reduce the probabilities of transmission to all nodes infected in the first epidemic by the factor $\alpha$ and obtain $\pi _{00}= p', \pi _{01}=\alpha p', \pi _{10}=p'$ and $\pi _{11}=\alpha p'$, as at (3.19). The proofs of the results corresponding to Theorems 3.1 and 3.2 then follow as above.

Turning to the final size of a large outbreak in the second epidemic, we derive now the conditional expectations in equations (6.10) and (6.11) for the offspring PGFs of the backward branching process $\mathcal {B}_B^{(2)}$. Consider first a vertex, *u* say, in the coloured tree $\mathcal {T}_*$, which is not the root. For $i \in \{1,2,3\}$, if *u* is of type *i* then the numbers of children of *u* of the three types in $\mathcal {T}_*$ is distributed as $(\tilde{Y}_{i1},\tilde{Y}_{i2},\tilde{Y}_{i3})$. Suppose that *u* is of type 1 or 2, i.e. that *u* was infected by the first epidemic. With probability $1-\alpha$, *u* is immune to the second epidemic and $\hat{X}_{i1}= \hat{X}_{i2}= \hat{X}_{i3}=0$. Otherwise each of *u*’s children in $\mathcal {T}_*$ contacts *u* in the second epidemic independently with probability $p'$. Hence, for $i \in \{1,2\}$,

$$\begin{aligned} &  \mathbb {E}[s_{1}^{\hat{X}_{i1} }s_{2}^{\hat{X}_{i2} } s_{3}^{\hat{X}_{i3} }|\tilde{Y}_{i1},\tilde{Y}_{i2},\tilde{Y}_{i3}]\\ &  \quad =1-\alpha +\alpha (1-p'+p's_1)^{\tilde{Y}_{i1}}(1-p'+p's_2)^{\tilde{Y}_{i2}}(1-p'+p's_3)^{\tilde{Y}_{i3}}. \end{aligned}$$

Thus, using (6.10),

$$\begin{aligned} \hat{f}_{i}(\textbf{s})=1-\alpha +\alpha f_{\tilde{\textbf{Y}}_i}(1-p'+p's_1,1-p'+p's_2,1-p'+p's_3)\quad (i \in \{1,2\}). \end{aligned}$$

Note the contrast with the forward branching process $\mathcal {B}_F^{(2)}$, in which each of *u*’s children is infected independently with probability that depends on their type (e.g. (6.5) and (6.6)). This underlines why the model with polarized partial immunity needs to be treated separately in the backward branching process $\mathcal {B}_B^{(2)}$ but not in the forward branching process $\mathcal {B}_F^{(2)}$. If *u* is of type 3, then it is necessarily susceptible to the second epidemic as it was not infected by the first epidemic. Again each of *u*’s children in $\mathcal {T}_*$ contacts *u* in the second epidemic independently with probability *p*, whence

$$\begin{aligned} \hat{f}_{3}(\textbf{s})=f_{\tilde{\textbf{Y}}_3}(1-p'+p's_1,1-p'+p's_2,1-p'+p's_3). \end{aligned}$$

Similar arguments for the initial generation show that, using (6.11),

$$\begin{aligned} \check{f}_1(\textbf{s})=1-\alpha +\alpha f_{\textbf{Y}_1}(1-p+ps_1,1-p+ps_2,1-p+ps_3) \end{aligned}$$

and

$$\begin{aligned} \check{f}_3(\textbf{s})=f_{\textbf{Y}_3}(1-p+ps_1,1-p+ps_2,1-p+ps_3). \end{aligned}$$

These all agree with the offspring PGFs given in the statement of Theorem 3.4, thus completing its proof.

## Extensions

In this paper we derive results for two sequential SIR epidemics on the same (static) configuration model network. Essential in our analysis is that the first epidemic is completely ended before the second epidemic starts. Furthermore, we assume that the infectious period of individuals is not random.

We think that it is not too hard to obtain similar results to those in this paper if we assume that the infectious periods of infected individuals are independent and identically distributed. However, we also recognise that it is not trivial. In particular, we will need a four-type branching process to approximate the epidemic instead of a three-type branching process in order to deal with dependencies which arise because whether or not two neighbours of a vertex (*v* say) are infected by the first epidemic both depend on the infectious period of *v* (Kuulasmaa 1982; Ball et al. 2009; Kenah and Miller 2011; Meester and Trapman 2011). To deal with random infectious periods, we need to consider $\vec {\mathcal {G}}$, the directed version of $\mathcal {G}$ in which all edges of $\mathcal {G}$ are replaced by two directed edges in opposite directions. The directed edges from an individual, *u* say, in $\vec {\mathcal {G}}$ are coloured red independently given *u*’s infectious period $I_u$, but with a probability that depends on $I_u$. Again we can approximate $\vec {\mathcal {G}}$ by a random tree ($\vec {\mathcal {T}}$ say), rooted at $w_0$, which is the initial infected individual for the second epidemic. We then colour a vertex in $\vec {\mathcal {T}}$ red if there is an infinite red path of edges directed towards it. Now assume that a given vertex, *v* say, is red because there is an infinite red path towards it, which does not involve the parent of *v* (so there is a red path of descent towards *v* in $\vec {\mathcal {T}}$). The main complication, not immediately solved by using just a directed network, is the following. Assume, in the terminology of Sect. 6.2, that *v* has a child which has no infinite red path from above towards it. Then whether or not this child is red is dependent on whether or not the parent of *v* is red, through the infectious period of *v*. We can deal with this dependency by assigning subtypes to type-1 vertices, in which vertex *v* is of type 1a if it has an infinite red path from above pointing towards it and there is a red edge from vertex *v* towards its parent. Vertex *v* is of type 1b if it has an infinite red path from above pointing towards it and there is no red edge from vertex *v* towards its parent. Just as in the fixed infectious period case, vertex *v* is of type 2 if it has at least one infinite red path towards it, but all those infinite red paths contain the parent of *v* as well, while a type-3 vertex is one which does not have a red path leading towards it. Once we have defined the four types of vertices in $\vec {\mathcal {T}}$, we expect the analysis of the second epidemic to be very similar in spirit to the analysis presented in this paper. However, the notation and presentation will become more cumbersome and will obscure the main message of this paper.

In the polarized partial immunity example we consider two repeated epidemics on the same graph $\mathcal {G}=(\mathcal {V},\mathcal {E})$, in which individuals infected in the first epidemic are susceptible to the second epidemic with probability $\alpha$ independently of one another. We might ask what happens if we have more than two epidemics and individuals which were either immune to the *k*-th epidemic ($k \in \mathbb {N}$), or were infected in the *k*-th epidemic are susceptible to the $k+1$-st epidemic with probability $\alpha$? After every epidemic the population is in a state in the state space $\{S,R\}^{\mathcal {V}}$ and those states after the epidemics form a Markov chain on $\{S,R\}^{\mathcal {V}}$ with a stationary distribution. It would be interesting, but probably hard, to analyse properties of this stationary distribution.

## References

[CR1] Andersson H (1999) Epidemic models and social networks. Math Sci 24(2):128–147

[CR2] Andersson H, Britton T (2000) Stochastic epidemic models and their statistical analysis. Springer, New York

[CR3] Ball F (2019) Susceptibility sets and the final outcome of collective Reed-Frost epidemics. Methodol Comput Appl Probab 21(2):401–421

[CR4] Ball F, Lyne O (2006) Optimal vaccination schemes for epidemics among a population of households, with application to variola minor in Brazil. Stat Methods Med Res 15(5):481–497 17089950 10.1177/0962280206071643

[CR5] Ball F, Neal P (2002) A general model for stochastic SIR epidemics with two levels of mixing. Math Biosci 180(1–2):73–102 12387917 10.1016/s0025-5564(02)00125-6

[CR6] Ball F, Sirl D, Trapman P (2009) Threshold behaviour and final outcome of an epidemic on a random network with household structure. Adv Appl Probab 41(3):765–796

[CR7] Ball F, Sirl D, Trapman P (2014) Epidemics on random intersection graphs. Ann Appl Probab 24(3):1081–1128. 10.1214/13-aap942

[CR8] Bansal S, Meyers LA (2012) The impact of past epidemics on future disease dynamics. J Theor Biol 309:176–184. 10.1016/j.jtbi.2012.06.012 22721993 10.1016/j.jtbi.2012.06.012

[CR9] Becker NG, Starczak DN (1998) The effect of random vaccine response on the vaccination coverage required to prevent epidemics. Math Biosci 154(2):117–135 9949651 10.1016/s0025-5564(98)10048-2

[CR10] Britton T (2010) Stochastic epidemic models: a survey. Math Biosci 225(1):24–35 20102724 10.1016/j.mbs.2010.01.006

[CR11] Britton T, Ball F, Trapman P (2020) A mathematical model reveals the influence of population heterogeneity on herd immunity to SARS-CoV-2. Science 369(6505):846–849 32576668 10.1126/science.abc6810 PMC7331793

[CR12] Britton T, Leung KY, Trapman P (2019) Who is the infector? General multi-type epidemics and real-time susceptibility processes. Adv Appl Probab 51(2):606–631

[CR13] Cox J, Durrett R (1988) Limit theorems for the spread of epidemics and forest fires. Stochast Processes Appl 30(2):171–191

[CR14] Diekmann O, Heesterbeek H, Britton T (2012) Mathematical tools for understanding infectious disease dynamics. Princeton University Press, Princeton

[CR15] Durrett R (2007) Random graph dynamics. Cambridge University Press, Cambridge

[CR16] Funk S, Jansen VA (2010) Interacting epidemics on overlay networks. Phys Rev E 81(3):036118 10.1103/PhysRevE.81.036118 20365826

[CR17] Grimmett G (1999) Percolation, 2nd edn. Springer, Heidelberg

[CR18] Halloran ME, Haber M, Longini IM Jr (1992) Interpretation and estimation of vaccine efficacy under heterogeneity. Am J Epidemiol 136(3):328–343 1415152 10.1093/oxfordjournals.aje.a116498

[CR19] Hardy GH, Littlewood JE, Pólya G (1952) Inequalities. Cambridge University Press, Cambridge

[CR20] van der Hofstad R (2016) Random graphs and complex networks vol. 1. Cambridge Series in Statistical and Probabilistic Mathematics, Cambridge

[CR21] Jacobsen KA, Burch MG, Tien JH, Rempała GA (2018) The large graph limit of a stochastic epidemic model on a dynamic multilayer network. J Biol Dyn 12(1):746–788 30175687 10.1080/17513758.2018.1515993

[CR22] Jagers P (1975) Branching processes with biological applications. Wiley, London

[CR23] Janson S (2014) The probability that a random multigraph is simple. II. J Appl Probab 51(A):123–137

[CR24] Kenah E, Miller JC (2011) Epidemic percolation networks, epidemic outcomes, and interventions. Interdiscip Perspect Infect Dis 2011(1):543520 21437002 10.1155/2011/543520 PMC3062991

[CR25] Kenah E, Robins JM (2007a) Network-based analysis of stochastic SIR epidemic models with random and proportionate mixing. J Theor Biol 249(4):706–722 10.1016/j.jtbi.2007.09.011 PMC2186204 17950362

[CR26] Kenah E, Robins JM (2007b) Second look at the spread of epidemics on networks. Phys Rev E 76(3):036113 10.1103/PhysRevE.76.036113 PMC2215389 17930312

[CR27] Kuulasmaa K (1982) The spatial general epidemic and locally dependent random graphs. J Appl Probab 19(4):745–758

[CR28] Lashari AA (2019) Stochastic epidemics on random networks. PhD thesis, Department of Mathematics, Stockholm University (2019)

[CR29] Meester R, Trapman P (2011) Bounding basic characteristics of spatial epidemics with a new percolation model. Adv Appl Probab 43(2):335–347

[CR30] Miller JC (2007) Epidemic size and probability in populations with heterogeneous infectivity and susceptibility. Phys Rev E 76(1):010101 10.1103/PhysRevE.76.010101 17677396

[CR31] Moore S, Mörters P, Rogers T (2018) A re-entrant phase transition in the survival of secondary infections on networks. J Stat Phys 171(6):1122–1135 31007280 10.1007/s10955-018-2050-9 PMC6445506

[CR32] Newman MEJ (2002) Spread of epidemic disease on networks. Phys Rev E 66(1):016128 10.1103/PhysRevE.66.016128 12241447

[CR33] Newman MEJ (2005) Threshold effects for two pathogens spreading on a network. Phys Rev Lett 95(10):108701 16196976 10.1103/PhysRevLett.95.108701

[CR34] Newman MEJ, Ferrario CR (2013) Interacting epidemics and coinfection on contact networks. PLoS ONE 8(8):71321 10.1371/journal.pone.0071321 PMC3738632 23951134

